# Well-Posedness Properties for a Stochastic Rotating Shallow Water Model

**DOI:** 10.1007/s10884-022-10243-1

**Published:** 2023-01-20

**Authors:** Dan Crisan, Oana Lang

**Affiliations:** Department of Mathematics, Imperial College London, London, UK

**Keywords:** Stochastic rotating shallow water models, SALT noise, Primary 60H15, secondary 60H30, 35R15, 35R60

## Abstract

In this paper, we study the well-posedness properties of a stochastic rotating shallow water system. An inviscid version of this model has first been derived in Holm (Proc R Soc A 471:20140963, 2015) and the noise is chosen according to the Stochastic Advection by Lie Transport theory presented in Holm (Proc R Soc A 471:20140963, 2015). The system is perturbed by noise modulated by a function that is not Lipschitz in the norm where the well-posedness is sought. We show that the system admits a unique maximal solution which depends continuously on the initial condition. We also show that the interval of existence is strictly positive and the solution is global with positive probability.

## Introduction

The rotating shallow water equations describe the evolution of a compressible rotating fluid below a free surface. The typical vertical length scale is assumed to be much smaller than the horizontal one, hence the *shallow* aspect. This model is a simplification of the primitive equations which are known for their complicated and computationally expensive structure (see e.g. [19]). Despite its simplified form, the rotating shallow water system retains key aspects of the atmospheric and oceanic dynamics [19, 37, 38]. It allows for gravity waves which play a highly important role in climate and weather modelling [37]. The classical inviscid shallow water model consists of a horizontal momentum equation and a mass continuity equation and in the presence of rotation it can be described as follows (see [13]):

$$\begin{aligned}{} & {} \epsilon \frac{D}{Dt}u_t + f\hat{z}\times u_{t}+\nabla p_{t} =0 \\{} & {} \quad \frac{\partial h_{t}}{\partial t}+\nabla \cdot \left( h_{t}u_{t}\right) =0 \end{aligned}$$

where

- $\frac{D}{Dt} := \frac{\partial }{\partial t} + u \cdot \nabla$ is the material derivative.
- $u=\left( u^1,u^2\right)$ is the horizontal fluid velocity vector field.
- $\epsilon$ is the Rossby number, a dimensionless number which describes the effects of rotation on the fluid flow: a small Rossby number ($\epsilon<<1$) suggests that the rotation dominates over the advective terms; it can be expressed as $\epsilon = \frac{U}{fL}$ where *U* is a typical scale for horizontal speed and *L* is a typical length scale.
- *f* is the Coriolis parameter, $f=2\Theta \sin \varphi$ where $\Theta$ is the rotation rate of the Earth and $\varphi$ is the latitude; $f\hat{z} \times u = \left( -fu^2, fu^1\right)$, where $\hat{z}$ is a unit vector pointing away form the centre of the Earth. For the analytical analysis we assume *f* to be constant.
- *h* is the thickness of the fluid column.
- $p := \frac{h-b}{\epsilon \mathscr {F}}$, $\nabla p$ is the pressure term, *b* is the bottom topography function.
- $\mathscr {F}$ is the Froude number, a dimensionless number which relates to the stratification of the flow. It can be expressed as $\mathscr {F}=\frac{U}{ NH}$ where *H* is the typical vertical scale and *N* is the buoyancy frequency.

The deterministic nonlinear shallow water equations (also known as the *Saint-Venant equations*) have been extensively studied in the literature. A significant difficulty in the well-posedness analysis of this model is generated by the interplay between its intrinsic nonlinearities, in the absence of any incompressibility conditions. In order to counterbalance the resulting chaotic effects, a viscous higher-order term is usually added to the inviscid system. Various shallow water models have been introduced for instance in [38] or [2]. In [26] the authors show global existence and local well-posedness for the 2D viscous shallow-water system in the Sobolev space $H^{s-\alpha }\left( \mathbb {R}^2\right) \times H^s\left( \mathbb {R}^2\right)$ with $s>1$ and $\alpha \in [0,1)$. The methodology is based on Littlewood-Paley approximations and Bony paraproduct decompositions. This extends the result in [27] where local solutions for any initial data and global solutions for small initial data have been obtained in $H^s \times H^s$ with $s>1$. A similar result adapted to Besov spaces was obtained in [28]. More recently, ill-posedness for the two-dimensional shallow water equations in critical Besov spaces has been shown in [24]. Existence of global weak solutions and convergence to the strong solution of the viscous quasi-geostrophic equation, on the two-dimensional torus is shown in [3]. In [4] the authors construct a sequence of smooth approximate solutions for the shalow water model obtained in [3]. The approximated system is proven to be globally well-posed, with height bounded away from zero. Global existence of weak solutions is then obtained using the stability arguments from [3]. Sundbye in [36] obtains global existence and uniqueness of strong solutions for the initial-boundary-value problem with Dirichlet boundary conditions and small forcing and initial data. In this work the solution is shown to be classical for a strictly positive time and a $C^0$ decay rate is provided. The proof is based on a priori energy estimates. Independently, Kloeden has shown in [20] that the Dirichlet problem admits a global unique and spatially periodic classical solution. Both [36] and [20] are based on the energy method developed by Matsumura and Nishida in [30]. Local existence and uniqueness of classical solutions for the Dirichlet problem associated with the non-rotating viscous shallow water model with initial conditions $(u_0,h_0) \in C^{2, \alpha } \times C^{1, \alpha }$ can be found in [6]. The proof is based on the method of successive approximations and Hölder space estimates, in a Lagrangian framework. Existence and uniqueness of solutions for the two-dimensional viscous shallow water system under minimal regularity assumptions for the initial data and with height bounded away from zero was proven in [7]. The possibly stabilising effects of the rotation in the inviscid case is analysed in [8, 29].

To simplify notation, we will denote by $a:=\left( v,h\right)$ the solution of the rotating shallow water (RSW) system and recast it in short form as^1^

$$\begin{aligned} da_{t}+F\left( a_{t}\right) dt =0, \end{aligned}$$

where $$F\left( a_{t}\right) $$ denotes

$$\begin{aligned} F\left( \begin{array}{c} v \\ h \end{array} \right) =\left( \begin{array}{c} u \cdot \nabla v + f\hat{z} \times u + \nabla p \\ \nabla \cdot (hu) \end{array} \right) \end{aligned}$$

where *u* is the fluid velocity and $v: =\epsilon u + \mathcal {R}$, with $curl \ \mathcal {R} = f\hat{z}$. $\mathcal {R}$ corresponds to the vector potential for the (divergence-free) rotation rate about the vertical direction, and it is chosen here such that $\nabla \mathcal {R} = 0$.

In this paper, we consider a viscous and stochastic version of the shallow water model described above, defined on the two-dimensional torus $\mathbb {T} ^{2}$:

1 $$\begin{aligned} da_{t}+F\left( a_{t}\right) dt +\sum _{i=1}^{\infty }\mathcal {G}_{i}\left( a_{t}\right) \circ dW_{t}^{i}=\gamma \Delta a_{t}dt, \end{aligned}$$

where $\gamma = (\nu ,\eta )$ is positive and corresponds to the fluid viscosity^2^, $W^{i}$ are independent Brownian motions, *F* is a nonlinear advective term, and $\mathcal {G}_{i}$ are differential operators explicitly described below. The integrals in (1) are of Stratonovitch type. The system (1) belongs to a class of stochastic models derived using the Stochastic Advection by Lie Transport (SALT) approach, as described in [17, 18, 35]. A detailed derivation of this specific system can be found in [22], following [17, 18]. In the stochastic case, *F* is defined as above, and

$$\begin{aligned} \mathcal {G}_{i}\left( \begin{array}{c} v \\ h \end{array} \right) =\left( \begin{array}{c} \mathcal {L}_i v + \mathcal {A}_i v \\ \mathcal {L}_i h \end{array} \right) \end{aligned}$$

where $\xi _{i}$ are divergence-free and time-independent vector fields, $\mathcal {L}_iv = \xi _i \cdot \nabla v$, $\mathcal {A}_iv = v^1\nabla \xi _i^1+v^2\nabla \xi _i^2$. The two operators $\mathcal {L}_i$ and $\mathcal {A}_i$ enjoy some properties which are described in Sect. 2 and in the Appendix. It has been shown lately that such stochastic parameters can be calibrated using data-driven approaches to account for the missing small-scale uncertainties which are usually present in the classical deterministic geophysical fluid dynamics models (see for instance [9, 10]). The addition of stochasticity in the advective part of the dynamics brings forth a more explicit representation of the uncertain transport behaviour in fluids, which draws on recent synergic advances in stochastic analysis, geophysical fluid dynamics, and data assimilation. The performance of these modern stochastic approaches is subject of intensive research. In [23] we have proven the applicability of this new stochastic model in a data assimilation framework.

To the best of our knowledge, this specific form of the stochastic rotating shallow water model has not been studied before. A stochastic version of the viscous rotating shallow water system with external forcing and multiplicative noise has been studied in [25]. This corresponds to the case $\mathcal {G}_{i}\left( a\right) =a$. A rotating shallow water model driven by Lévy noise has been considered in [14]. As pointed out in [25], the number of results available in the literature on stochastic shallow water equations is limited. In the deterministic case, existence of solutions under certain conditions and without rotation was proven in [31]. Smooth approximate solutions for the 2D deterministic rotating shallow water system have been constructed in [5]. Long time existence for rapidly rotating deterministic shallow water and Euler equations has been shown in [8].

### Contributions of the Paper

The first contribution of the paper is the existence of local solutions $(a,\tau )$ of the system (1) with paths $t\rightarrow a_{t\wedge \tau }$ in the space^3^

$$\begin{aligned} C\left( [0,T],\mathcal {W}^{1,2}\left( \mathbb {T}^2\right) ^{3}\right) \cap L^{2}\left( 0,T;\mathcal {W}^{2,2}\left( \mathbb {T}^2\right) ^3\right) ,~\ ~T>0 \end{aligned}$$

provided the initial datum $a_{0}\in \left( \mathcal {W}^{1,2}\left( \mathbb {T}^2\right) \right) ^{3},$ where $\tau$ is a strictly positive stopping time specified below. Subsequently, we prove that there exists a unique strong maximal solution $(a,\tau _{\textrm{max }})$ of the system (1), see Theorem 3.1 below. We also show that the solution depends continuously on the initial data, see Theorem 3.8 below.

The structure of the noise studied here is different from that in [25], in particular the operators ${\mathcal {G}}_i$ are not Lipschitz. As a result, we need to use a different approximation method, extending the methodology developed in [11] and [12] to a system of SPDEs which model a compressible fluid under the effects of rotation. In particular, we do not use Galerkin approximations as in [25]. Instead, we construct a sequence that approximates a truncated version of the equation, where the nonlinear term is replaced by a forcing term depending on the previous element of the sequence. The sequence is well defined by using a classical result of Rozovskii (see Theorem 2, pp. 133, in [33]).

The main proof of existence of a truncated solution (see Theorem 3) builds upon the arguments developed in [11, 12], where the analysis of the vorticity version of the fluid equation was accomplished. This way, the pressure term has been eliminated altogether and the equation has been closed by expressing the velocity vector field in terms of the vorticity via a Biot-Savart operator (see e.g. [11] for details). This is no longer possible here: the equation satisfied by the fluid vorticity contains a term that depends on $\nabla p$, where $p=\frac{h-b}{ \epsilon \mathscr {F}}.$ This implies on the one hand that an $L^{p}$ -transport property for the vorticity is out of reach, and also that a control of the higher order derivatives is much more difficult.

As it is well known, compressible systems are much harder to analyse than their incompressible counterparts. In the particular case of (1 ), the pressure term $p=\frac{h-b}{\epsilon \mathscr {F}}$ persists in all the a priori estimates. To be more precise, the inner product $\langle v,\nabla p\rangle$ does not vanish as it is the case for incompressible systems. Obviously, the same is true for the higher derivatives $\langle \partial ^{\alpha }v,\partial ^{\alpha }\nabla p\rangle$. The control of the nonlinear terms is no longer possible in the same manner as in the incompressible case. As a result, without adding extra viscosity we cannot “close” any inequalities involving Sobolev norms of *v* and *h*: to control $\left| \left| v\right| \right| _{1,2},$ we need a control of $\left| \left| h\right| \right| _{2,2}$ which requires $\left| \left| v\right| \right| _{2,2}~$and so on in a never-ending procession. In spectral theory language, there is an energy cascade between low frequency and high frequency modes. To balance the equation and close the loop, we add viscosity to the system. This way, energy dissipates from all modes and we succeed to show that, at least for a while, the solution does not blow-up.

The stochasticity incurs additional technical difficulties. That is because the solution might blow-up at some random time $\tau _{blow-up}$. For any deterministic time $t>0$, $\tau _{blow-up}$ might be larger than *t* for some realizations of the noise, in other words the solution blows up after time *t* if at all, whilst for other realizations of the noise, $\tau _{blow-up}$ might be smaller than *t*, in other words the solution blows up before time *t*. As a result, the expectations of the random variables appearing in (1) might not exist. In particular, we cannot prove that

2 $$\begin{aligned} \mathbb {E}\left[ \left| \left| a_{t}\right| \right| _{1,2}^{2}\right]<\infty ,~~\mathbb {E}\left[ \int _{0}^{t}\left| \left| a_{t}\right| \right| _{2,2}^{2}ds\right]<\infty ,\sum _{i=1}^{\infty }\int _{0}^{t}\mathbb {E}\left[ \left| \left| \mathcal {G}_{i}\left( a_{s}\right) \right| \right| _{2}^2\right] ds<\infty , \end{aligned}$$

etc. or any other suitable controls of expectations. In particular, the Doob-Meyer decomposition of the semi-martingale process $t\rightarrow a_{t}$ contains a *local* martingale. On the technical side, the standard (deterministic) Gronwall approach cannot be used to control the Sobolev norm of the solution $t\rightarrow a_{t}$ of (1) pathwise (because of the stochasticity), neither can a control of $t\rightarrow \mathbb {E}\left[ \left| \left| a_{t}\right| \right| _{1,2}^{2}\right]$ be deduced (because of the possibility of finite blow-up). However, if we “stop” the process at a time $\tau$ that is *sure to occur* before the blow-up, $\tau <\tau _{blow-up}$, then we *can* obtain controls on expectations such as the ones appearing in (2).

The definition of the solution of (1) plays a crucial role in the analysis, more so than in the deterministic case, and we introduce it in the next section.

In the absence of noise, one can prove that the viscous RSW has a global solution for sufficiently small initial datum, $\left| \left| a_{0}\right| \right| \le \varepsilon$, where $\varepsilon =\varepsilon \left( \gamma \right)$. That is because one can show that there exist constants $b=b\left( \gamma \right)$ and $c=c\left( \gamma \right)$ such that

$$\begin{aligned} {\textrm{d}}_{\textrm{t}}\left| \left| a\right| \right| _{1,2}^{2}\le r_{b,c} \left| \left| a\right| \right| _{1,2}^{2} , \end{aligned}$$

where $\left| \left| \cdot \right| \right| _{1,2}$ is the Sobolev norm of the space $\left( \mathcal {W}^{1,2}\left( \mathbb {T}^2\right) \right) ^{3}$and $r_{b,c}:[0,\infty )\rightarrow [0,\infty ), \ r_{b,c}\left( s\right) =bs^{3}-cs$. The reason for this is that the solution of the ODE $d_{t}q=r_{b,c}\left( q\right)$ is an upper bound for $t\rightarrow \left| \left| a_{t}\right| \right| _{1,2}^{2}.$ The function $t\rightarrow q_{t}$ is bounded if the initial condition belongs to the interval $\ [0,\sqrt{c/b}]$. In fact, $\lim _{t\rightarrow \infty }q_{t}=0$ if the initial condition belongs to the interval $\ [0,\sqrt{c/b})$ and it is constant if $$q_{0}=\sqrt{c/b}.\ $$However, if $q_{0}>\sqrt{c/b}$ then the solution blows up in finite time. This does not necessarily mean that the solution of the SRSW model blows up in finite time. In the stochastic case, we can deduce a corresponding *stochastic* differential equation that gives us an upper bound $\left| \left| a\right| \right| _{1,2}^{2}$. The solution of this can be shown to remain bounded only with positive probability.

Therefore, we can prove that $\mathbb {P}\left( \tau _{blow-up}=\infty \right) >0$, but not that $\mathbb {P}\left( \tau _{blow-up}=\infty \right) =1$. In fact, it is not necessarily true that the solution will actually blow up if it is global: we prove that the solution *remains* uniformly bounded on $[0,\infty )$, not just that it does not blow up in finite time. The result we obtain gives only a sufficient condition for global existence. In future work we aim to show that, under suitable additional assumptions on the choice of the noise and on that of the initial condition the stochastic RSW equation exists *globally with probability 1*.

Similar results (and similar proofs) hold for $a_{0}\in \mathcal {W}^{k,2}\left( \mathbb {T}^2\right) ^{3}, \ k>1$. In this case, the system (1) has paths $t\rightarrow a_{t\wedge \tau }$ in the space

$$\begin{aligned} C\left( [0,T],\left( \mathcal {W}^{k,2}\left( \mathbb {T}^2\right) \right) ^{3}\right) \cap L^{2}\left( 0,T;\left( \mathcal {W}^{k+1,2}\left( \mathbb {T}^2\right) \right) ^{3}\right) ,~\ ~T>0, \end{aligned}$$

and the maximal time can be characterized in a similar manner to the classical Beale-Kato-Majda criterion. The justification of such criteria is the subject of future work.

### Structure of the Paper

We construct sequences of approximating solutions which converge in a suitable sense to a truncated form of the original SRSW system. Then we show that the truncation can be lifted. As opposed to the case of the Euler equation, here we can lift the truncation only up to a positive stopping time. This is due to a lack of transport properties for both variables, which derive from the compressibility condition and the form of the nonlinear terms. Therefore we obtain a local solution for the original system (3). This is proven in Sect. 3. In Sect. 3.1 we show that this solution is maximal and due to the pathwise uniqueness property from Sect. 3.2, it is also strong in probabilistic sense. In Sect. 3.3 we show global existence for the truncated model. In Sect. 5 we study the analytical properties of the approximating sequence. A couple of a priori estimates and other useful results are presented in the Appendix.

## Preliminaries and Notations

In this section we introduce the main notations, together with the Itô form of the system, definition of solutions and other assumptions and remarks.

### Notations

- As mentioned above, we work on the two-dimensional torus denoted by $\mathbb {T}^{2}$.
- Let $\left( \Omega ,\mathcal {F},\left( \mathcal {F}_{t}\right) _{t},\mathbb {P},\left( W^{i}\right) _{i}\right)$ be a fixed stochastic basis consisting of the filtered probability space $\left( \Omega ,\mathcal {F},\left( \mathcal {F}_{t}\right) _{t},\mathbb {P}\right)$ and a sequence of independent one-dimensional Brownian motions $\left( W^i\right) _i$ which are adapted to the complete and right-continuous filtration $\left( \mathcal {F}_t\right) _t$.
- Let $\mathcal {M}_t:=L^2\left( \Omega , C\left( [0,t];\mathcal {W}^{1,2}\left( \mathbb {T}^2\right) ^3\right) \right) \cap L^2\left( \Omega , L^2\left( 0,t;\mathcal {W}^{2,2}\left( \mathbb {T}^2\right) ^3\right) \right)$ for all $t \ge 0$. For a stochastic process *b* belonging to the space $\mathcal {M}_t$ we define the norm
  $$\begin{aligned} \Vert b\Vert _{t,2,2}^2 := \displaystyle \sup _{s\in [0,t]}\Vert b_s\Vert _{1,2}^2 + \displaystyle \int _0^t\Vert b_s\Vert _{2,2}^2ds. \end{aligned}$$
  Define also
  $$\begin{aligned} {\left| \left| \left| a_t \right| \right| \right| } :=\sup _{s\in \left[ 0,t\right] }\left( \Vert v_{s}\Vert _{1,2}^{2}+\Vert h_{s}\Vert _{1,2}^{2}\right) . \end{aligned}$$
  It follows that $a, b \in \mathcal {M}_{t}$ implies $\mathbb {E}[\Vert b\Vert _{t,2,2}] < \infty$ and $\mathbb {E}\left[ {\left| \left| \left| a_t \right| \right| \right| }\right] <\infty$ for all $t>0$.
- Let $\alpha \in (0,1), p\in [2,\infty )$ and let *H* be a Hilbert space. Then the fractional Sobolev space $\mathcal {W}^{\alpha ,p}(0,T; H)$ is endowed with the norm
  $$\begin{aligned} \Vert f\Vert _{\mathcal {W}^{\alpha , p}(0, T ; H)}^p:=\int _{0}^{T}\left\| f_{t}\right\| _{H}^{p} d t+\int _{0}^{T} \int _{0}^{T} \frac{\left\| f_{t}-f_{s}\right\| _{H}^{p}}{|t-s|^{1+\alpha p}} d t d s. \end{aligned}$$
- *C* is a generic constant and can differ from line to line.

### Itô form and Definition of Solutions

The expanded version of the stochastic system (1) is

3a $$\begin{aligned}{} & {} dv_{t}+\big [u_{t}\cdot \nabla v_{t}+f\hat{z}\times u_{t}+\nabla p_{t}\big ]dt+ \displaystyle \sum _{i=1}^{\infty }\big [\left( \mathcal {L}_{i}+\mathcal {A}_{i}\right) v_{t} \big ]\circ dW_{t}^{i}=\nu \Delta v_{t}dt \end{aligned}$$

3b $$\begin{aligned}{} & {} \quad dh_{t}+\nabla \cdot (h_{t}u_{t})dt+\displaystyle \sum _{i=1}^{\infty }\big [ \nabla \cdot \left( \xi _{i}h_{t}\right) \big ]\circ dW_{t}^{i}=\eta \Delta h_{t}dt. \end{aligned}$$

The corresponding Itô form of the system (3) is given below

4a $$\begin{aligned} dv_t&+ \big [\mathcal {L}_{u_t} v_t + f\hat{z} \times u_t + \nabla p_t - \nu \Delta v_t\big ]dt \nonumber \\&+ \displaystyle \sum _{i=1}^{\infty }\big [(\mathcal {L}_i + \mathcal {A}_i)v_t\big ] dW_t^i = \frac{1}{2}\displaystyle \sum _{i=1}^{\infty } \big [\left( \mathcal {L}_i + \mathcal {A}_i\right) ^2v_t\big ]dt \end{aligned}$$

4b $$\begin{aligned}&\quad dh_t+ \big [\nabla \cdot (h_tu_t) - \eta \Delta h_t\big ]dt+\displaystyle \sum _{i=1}^{\infty }\mathcal {L}_ih_t dW_t^i=\frac{1}{2}\displaystyle \sum _{i=1}^{\infty }\mathcal {L}_i^2h_tdt. \end{aligned}$$

In the following, we will work with the Itô version (4) of the system (3). The definition of a solution of the system (4) is made explicit in Definition 2.1 below. Using the Itô version (4) of the system as basis for the well-posedness analysis enables us to match the constraints imposed on the initial condition to guarantee the well-posedness of the deterministic system. In particular, Theorems 3.1 and 3.2 below state that the system (4) is well-posed provided $a_0=(v_0,h_0)\in \left( W^{1,2}\left( \mathbb {T}^2\right) \right) ^3$.

The required smoothness constraint on $a_0$ that ensures the existence of a strong solution for the equation *written in Stratonovitch form*, i.e. the system (3), is $a_0=(v_0,h_0)\in \left( W^{1,2}\left( \mathbb {T}^2\right) \right) ^3$ . Let us explain why: the Stratonovich integrals in the system (3) require that the integrands are semi-martingales, in this case, the processes $\mathcal {G}_{i} a$. The evolution equation for the processes $\mathcal {G}_{i} a_s$ involves the terms $\mathcal {G}_{i} ^{3} a_s$ which make sense if the paths of the solution are in $L^2 \left( [0,T], W^{3,2}\left( \mathbb {T}^2\right) \right)$. This can be achieved, due to the added viscosity term, if the initial condition $a_0\in \left( W^{2,2}\left( \mathbb {T}^2\right) \right) ^3$. To avoid the additional smoothness requirement we work with the Itô version (4) of the system.

We introduce the following notions of solutions:

#### Definition 2.1

- A pathwise local solution of the SRSW system is given by a pair $(a,\tau )$ where $\tau :\Omega \rightarrow [0,\infty ]$ is a strictly positive bounded stopping time, $a_{\cdot \wedge \tau }:\Omega \times [0,\infty )\times \mathbb {T}^{2}\rightarrow \mathbb {R}^{3}$ is an $\mathcal {F}_{t}$-adapted process for any $t\ge 0$, with initial condition $a_{0}$, such that
  $$\begin{aligned} a_{\cdot \wedge \tau }\in L^{2}\left( \Omega ;C\left( [0,T];\mathcal {W} ^{1,2}\left( \mathbb {T}^{2}\right) ^{3}\right) \right) \cap L^{2}\left( \Omega ;L^{2}\left( 0,T;\mathcal {W}^{2,2}\left( \mathbb {T}^{2}\right) ^{3}\right) \right) \end{aligned}$$
  and the SRSW system (1) is satisfied locally i.e.
  5 $$\begin{aligned} a_{t\wedge \tau }=a_{0}+\int _{0}^{_{t\wedge \tau }}\tilde{F}(a_{s}) ds - \sum _{i=1}^{\infty }\int _{0}^{_{t\wedge \tau }}\mathcal {G}_{i}(a_{s}) dW_{s}^{i} + \gamma \int _{0}^{_{t\wedge \tau }}\Delta a_{s}ds, \end{aligned}$$
  holds $\mathbb {P}$-almost surely, as an identity in $L^{2}\left( \mathbb {T}^{2}\right) ^{3},$ with $\tilde{F}(a_s):= - F(a_s) + \frac{1}{2}\displaystyle \sum _{i=1}^{\infty } \mathcal {G}_i^2(a_s)$.
- If $\tau =\infty$ then the solution $a=(v,h)$ is called global.
- A pathwise maximal solution of the SRSW system is given by a pair $(a, \mathcal {T})$ where $\mathcal {T}: \Omega \rightarrow [0, \infty ]$ is a non-negative stopping time and $a=(a_{t\wedge \mathcal {T}})_t$, $a_{t\wedge \mathcal {T}}: \Omega \times \mathbb {T}^2 \rightarrow \mathbb {R}^3$ is a process for which there exists an increasing sequence of stopping times $(\tau ^n)_n$ with the following properties:
  i. $$\mathcal {T}=\lim _{n\rightarrow \infty }\tau ^{n}$$ and $$\mathbb {P}( \mathcal {T}>0)=1$$
  ii. $\left( a,\tau ^{n}\right)$ is a pathwise local solution of the SRSW system for every $n\in \mathbb {N}$
  iii. if $\mathcal {T}<\infty$ then
    $$\begin{aligned} \displaystyle \limsup _{t \rightarrow \mathcal {T}} \Vert a_{t}\Vert _{1,2} = \infty . \end{aligned}$$
- A weak/martingale local solution of the SRSW system is given by a triple $\left( \left( \check{a},\left( \check{W}^{i}\right) _{i}\right) ,\left( \check{\Omega },\mathcal {\check{F}},\check{ \mathbb {P}}\right) ,\left( \mathcal {\check{F}}_{t}\right) _{t}\right)$ such that $\left( \check{\Omega }, \mathcal {\check{F}},\left( \mathcal {\check{F}}_{t}\right) _{t},\check{\mathbb {P}},\left( \check{ W}^{i}\right) _{i}\right)$ is a stochastic basis, $\check{a}$ is a continuous $(\mathcal { \check{F}}_{t})_{t}$-adapted real valued process, $\check{a}: \check{\Omega } \times \mathbb {T}^{2}\rightarrow \mathbb {R}^{3}$, which satisfies (1) for a stopping time $\tau :\check{\Omega }\rightarrow [0,\infty ]$, $\left( \check{W}^{i}\right) _{i}$ are independent $\left( \mathcal {\check{F}}_{t}\right) _{t}$ -adapted Brownian motions, and all identities hold $\check{\mathbb {P}}$ -almost surely in $L^{2}\left( \mathbb {T}^{2}\right) ^{3}$.

#### Remark 2.2

Note that throughout the paper, the space of solution is

$$\begin{aligned} L^{2}\left( \Omega ;C\left( [0,T];\mathcal {W} ^{1,2}\left( \mathbb {T}^{2}\right) ^{3}\right) \right) \cap L^{2}\left( \Omega ;L^{2}\left( 0,T;\mathcal {W}^{2,2}\left( \mathbb {T}^{2}\right) ^{3}\right) \right) . \end{aligned}$$

By this, we mean that

$$\begin{aligned}{} & {} a_{\cdot \wedge \tau }\in L^2\left( \Omega , C\left( [0,T];\mathcal {W}^{1,2}\left( \mathbb {T}^2\right) ^3\right) \right) \\{} & {} \quad a\mathbb {1}_{t\le \tau } \in L^2\left( \Omega , L^2\left( 0, T;\mathcal {W}^{2,2}(\mathbb {T}^2)^3\right) \right) . \end{aligned}$$

#### Remark 2.3

- We will show below that the SRSW system (1) satisfies the local uniqueness property. In other words, if $\left( a^1,\tau ^1\right)$ and $\left( a^2,\tau ^2\right)$ are two local solutions to system (1), then they must coincide on the interval $\left[ 0,\tau ^1\wedge \tau ^2\right]$. Using the local uniqueness property, we will deduce that a stopping time $\mathcal {T}$ satisfying property iii. of the definition above is the largest stopping time with properties *i*. and *ii*., that is for any other pair $\left( a^{\prime },\mathcal {T}^{\prime }\right)$ which satisfies *i*. and *ii*. we necessarily have $\mathcal {T}^{\prime }\le \mathcal {T}$ $\mathbb {P}$-a.s., and $a=a^{\prime }$ on $[0,\mathcal {T}^{\prime }]$.
- The first two definitions of solutions are established with respect to a fixed stochastic basis, the solutions being *strong* in probabilistic sense. The solution defined at d. is weak in probabilistic sense, meaning that $\check{a} = \left( \check{v}, \check{h}\right)$ is not necessarily adapted to the original filtration $\left( \mathcal {F}_t\right) _t$ generated by the driving Brownian motion which corresponds to the SRSW system (3).

### Assumptions and remarks

The vector fields $\xi _i:\mathbb {T}^2 \rightarrow \mathbb {R} ^2$ are chosen to be time-independent and divergence-free, such that

6 $$\begin{aligned} \displaystyle \sum _{i=1}^{\infty } \Vert \xi _i\Vert _{4,\infty }^2 < \infty . \end{aligned}$$

Condition (6) implies that the infinite sums of stochastic integrals

7 $$\begin{aligned} \sum _{i=1}^{\infty } \int _0^t \mathcal {G}_{i} (a_s)dW_s^i, \ \ \sum _{i=1}^{\infty } \int _0^t \nabla \mathcal {G}_{i} (a_s)dW_s^i \end{aligned}$$

are well defined and belong to $L^2\left( 0, T; L^2\left( \mathbb {T}^2\right) ^3\right)$, provided the process $s\rightarrow a_s$ has paths in the space $L^2\left( 0,T; \mathcal {W}^{2,2}\left( \mathbb {T}^2\right) ^3\right)$ for $T\ge 0$. Local solutions of the SRSW model as defined above have this property. Similarly, the infinite sums of the Riemann-Stieltjes integrals

8 $$\begin{aligned} \sum _{i=1}^{\infty } \int _0^t \mathcal {G}^2_{i} (a_s)ds, \end{aligned}$$

are well-defined and belong to $L^2\left( 0, T; L^2\left( \mathbb {T}^2\right) ^3\right)$.

## Existence and Uniqueness of Strong Pathwise Solutions for the SRSW System

In this section we present the main results of this paper.

### Theorem 3.1

Let $\mathcal {S} = \left( \Omega , \mathcal {F}, \left( \mathcal {F}_t\right) _t, \mathbb {P}, \left( W^i\right) _i\right)$ be a fixed stochastic basis and $a_0\in \mathcal {W} ^{1,2}(\mathbb {T}^2)^3$. Then the stochastic rotating shallow water system (3) admits a unique pathwise maximal solution $(a,\mathcal {T})$ which belongs to the space

$$\begin{aligned}L^{2}\left( \Omega ;C\left( [0,\mathcal {T});\mathcal {W}^{1,2}\left( \mathbb {T}^{2}\right) ^{3}\right) \right) \cap L^{2}\left( \Omega ;L^{2}\left( 0,\mathcal {T};\mathcal {W}^{2,2}\left( \mathbb {T}^{2}\right) ^{3}\right) \right) .\end{aligned}$$

The existence of a solution for system (3) is proved by first showing that a truncated version of it has a solution and then removing the truncation up to a positive stopping time. In particular, we truncate the nonlinear terms in (3) using a smooth function $f_R: \mathbb {R}_{+} \rightarrow [0,1]$ equal to 1 on [0, *R*], equal to 0 on $[R+1, \infty )$, and decreasing on $[R, R+1]$, $f_{R}\left( a_{t}\right) :=f_{R}\left( \Vert a_t\Vert _{1,2}\right)$ where $\Vert a_t\Vert _{1,2}:=\Vert v_t\Vert _{1,2}+\Vert h_{t}\Vert _{1,2}$ for arbitrary $R>0$. The choice of the truncation $f_{R}\left( a_{t}\right)$ is such that the nonlinear terms are uniformly bounded *pathwise* in $L^{2}\left( 0,T;L^{2}\left( \mathbb {T} ^{2}\right) ^3\right)$ for any $T\ge 0$. Then we have the following:

### Theorem 3.2

Let $\mathcal {S} = \left( \Omega , \mathcal {F}, (\mathcal {F} _t)_t, \mathbb {P}, \left( W^i\right) _i\right)$ be a fixed stochastic basis and

$a_0\in \mathcal {W }^{1,2}\left( \mathbb {T}^2\right) ^3$. Then the truncated system

9a $$\begin{aligned}&dv_{t}^R+\bigg [f_{R}\left( a_{t}^R\right) \mathcal {L}_{u_{t}^R}v_{t}^R+f\hat{z}\times u_{t}^R+ \nabla p_{t}^R - \nu \Delta v_{t}^R\bigg ]dt\nonumber \\&+\displaystyle \sum _{i=1}^{\infty }\big [\left( \mathcal {L}_{i}+\mathcal {A}_{i}\right) v_{t}^R\big ]\circ dW_{t}^{i}=0 \end{aligned}$$

9b $$\begin{aligned}&\quad dh_{t}^R+\bigg [f_{R}\left( a_{t}^R\right) \nabla \cdot \left( h_{t}^Ru_{t}^R\right) - \eta \Delta h_{t}^R\bigg ]dt+ \displaystyle \sum _{i=1}^{\infty }\big [\nabla \cdot \left( \xi _{i}h_{t}^R\right) \big ]\circ dW_{t}^{i}=0 \end{aligned}$$

admits a unique global pathwise solution $a^{R}=\left\{ \left( v_{t}^{R},h_{t}^{R}\right) ,t\in [0,\infty )\right\}$ such that

$$\begin{aligned} a^{R}\in L^{2}\left( \Omega ;C\left( [0,T];\mathcal {W}^{1,2}(\mathbb {T} ^{2})^{3}\right) \right) \cap L^{2}\left( \Omega ;L^{2}\left( 0,T;\mathcal {W }^{2,2}(\mathbb {T}^{2})^{3}\right) \right) \end{aligned}$$

for any $$T>0$$.

Theorem 3.2 is proved in Sect. 3.3.

Define

10 $$\begin{aligned} \tau ^{R}:=\inf _{t\ge 0}\left\{ \Vert a_t\Vert _{1,2}\ge R\right\} . \end{aligned}$$

### Proposition 3.3

Given $a_0 \in \left( \mathcal {W}^{1,2}\left( \mathbb {T}^2\right) \right) ^3$ and $R>0$, the restriction $a: \Omega \times [0, \tau ^R) \times \mathbb {T}^2 \rightarrow \mathbb {R}^3$ of the global solution $a : \Omega \times [0, \infty ) \times \mathbb {T}^2 \rightarrow \mathbb {R}^3$ corresponding to the truncated system (9) is a local solution of the original SRSW system (3).

### Proof

For $t\in [0, \tau ^R]$ $f_R\left( a^R\right) =1$ therefore the truncated system (9) and the original SRSW system (3) coincide. $\square$

### Maximal Solution for the SRSW System

#### Proposition 3.4

Given $a_0\in \mathcal {W}^{1,2}\left( \mathbb {T}^2\right) ^3$ and $R>0$, there exists a unique maximal solution $\left( a,\mathcal {T}\right)$ of the original SRSW system (3) such that

11 $$\begin{aligned} \displaystyle \limsup _{t\rightarrow \mathcal {T}}\left| \left| a_t\right| \right| _{1,2}=\infty , \end{aligned}$$

whenever $$\mathcal {T}<\infty $$.

#### Proof

**Existence.** If we choose $$R=n$$ in Proposition 3.3 then $$\left( a^{n},\tau ^{n}\right) $$ is a local solution of the SRSW system (3). Moreover, observe that $$ a^{n+1}$$ satisfies Eq. (9) for $$R=n$$ on the interval $$[0,\tau ^{n}]$$. By the local uniqueness, it follows that

12 $$\begin{aligned} a^{n+1}|_{[0,\tau ^{n}]}=a^{n}|_{[0,\tau ^{n}]}. \end{aligned}$$

Define $$\mathcal {T}:=\displaystyle \lim _{n\rightarrow \infty }\tau ^{n}$$ and

13 $$\begin{aligned} a|_{[0,\tau ^{n}]}:=a^{n}|_{[0,\tau ^{n}]}. \end{aligned}$$

Definition (13) is consistent following (12). It only remains to show (11). If $\displaystyle \lim _{n\rightarrow \infty }\tau ^{n}=\mathcal {T}<\infty$ then

$$\begin{aligned} \displaystyle \limsup _{t\rightarrow \mathcal {T}}\left| \left| a_t\right| \right| _{1,2}\ge \displaystyle \limsup _{n\rightarrow \infty }\Vert a_{\tau _n}\Vert _{1,2} = \displaystyle \limsup _{n\rightarrow \infty } n = \infty . \end{aligned}$$

**Uniqueness**. Assume that $\left( \bar{a}{,\mathcal {\bar{T}}}\right)$ is another solution with $\left( \bar{a},\bar{\tau }_n \right)$, $n\ge 1$ being the corresponding sequence of local solutions converging to the maximal solution. By the uniqueness of the truncated equation it follows that $\bar{a}=a$ on $[0,\bar{\tau }^{n}\wedge \tau ^{n}]$ . By taking the limit as $n\rightarrow \infty$ it follows that $\bar{a}=a$ on $[0,\mathcal {T}\wedge \mathcal {\bar{T}})$. We prove next that $\mathcal {T} =\mathcal {\bar{T}}$, $\mathbb {P}-a.s.$. Let us assume that

$$\begin{aligned} \mathbb {P}\left( \omega \in \Omega ,\mathcal {T}\left( \omega \right) \ne \mathcal {\bar{T}}\left( \omega \right) \right) >0. \end{aligned}$$

Observe that

$$\begin{aligned} \mathbb {P}\left( \omega \in \Omega ,\mathcal {T}\left( \omega \right) \ne \mathcal {\bar{T}}\left( \omega \right) \right) \le \mathbb {P}\left( \Xi ^{1}\right) +\mathbb {P}\left( \Xi ^{2}\right) , \end{aligned}$$

where

$$\begin{aligned} \Xi ^{1}= & {} \left\{ \omega \in \Omega ,\mathcal {T}\left( \omega \right)<\infty ,\mathcal {T}\left( \omega \right)<\mathcal {\bar{T}}\left( \omega \right) \right\} , \\ \Xi ^{2}= & {} \left\{ \omega \in \Omega ,\mathcal {\bar{T}}\left( \omega \right)<\infty ,\mathcal {\bar{T}}\left( \omega \right) <\mathcal {T}\left( \omega \right) \right\} . \end{aligned}$$

We prove that $\mathbb {P}\left( \Xi ^{1}\right) =\mathbb {P}\left( \Xi ^{2}\right) =0$. The two sets are symmetric so we show this only for the first one. From the definition of the local solution, observe that

$$\begin{aligned} \mathbb {E}\left[ \sup _{s\in [0,\bar{\tau }^{n}\left( \omega \right) ]}\Vert \bar{a}_t\Vert _{1,2}\right] <\infty \end{aligned}$$

hence

$$\begin{aligned} \mathbb {P}\left( \omega \in \Omega ,\sup _{s\in [0,\bar{\tau } ^{n}\left( \omega \right) ]}\Vert \bar{a}_t\Vert _{1,2}=\infty \right) =0. \end{aligned}$$

However, if $\mathcal {T}\left( \omega \right) <\infty$ and $\mathcal {T} \left( \omega \right) <\bar{\tau }^{n}\left( \omega \right)$ then

$$\begin{aligned} \infty =\sup _{s\in [0,\mathcal {T}\left( \omega \right) )}\Vert a_s\Vert _{1,2}=\sup _{s\in [0,\mathcal {T}\left( \omega \right) )}\Vert \bar{a}_s\Vert _{1,2}\le \sup _{s\in [0,\bar{\tau }^{n}\left( \omega \right) ]}\Vert \bar{a}_s\Vert _{1,2}. \end{aligned}$$

It follows that $\mathbb {P}\left( \omega \in \Omega ,\mathcal {T}\left( \omega \right)<\infty ,\mathcal {T}\left( \omega \right) <\bar{\tau } ^{n}\left( \omega \right) \right) =0.$ Hence

$$\begin{aligned} \mathbb {P}\left( \Xi ^{1}\right) =\lim _{n\rightarrow \infty }\mathbb {P} \left( \omega \in \Omega ,\mathcal {T}\left( \omega \right)<\infty ,\mathcal { T}\left( \omega \right) <\bar{\tau }^{n}\left( \omega \right) \right) =0. \end{aligned}$$

This completes the proof of the uniqueness claim. $\square$

The purpose of the next proposition is to show that the maximal solution constructed in Proposition 3.4 has paths in $L^{2,loc}\left( [0,\mathcal {T}), \left( \mathcal {W}^{2,2}\left( \mathbb {T}^2\right) \right) ^3\right)$. Recall the definition of $\tau ^N$ as given in (10) and the definition of $\Vert a\Vert _{t,2,2}$ as introduced in (2.1) and introduce a new sequence of stopping times $\left( \hat{\tau }^M\right) _M$ with

$$\begin{aligned} \hat{\tau }^M := \displaystyle \inf _{t\ge 0}\{ \Vert a\Vert _{t,2,2} \ge M\}. \end{aligned}$$

Define $$\hat{\mathcal {T}} := \displaystyle \lim _{M\rightarrow \infty } \hat{\tau }^M$$.

#### Corollary 3.5

Let $(a, \mathcal {T})$ be the maximal solution constructed in Proposition 3.4. Then the process $t\rightarrow a_{t\wedge \tau ^R}$ takes values in

$$\begin{aligned} L^{2}\left( \Omega ;C\left( [0,T];\mathcal {W}^{1,2}\left( \mathbb {T} ^{2}\right) ^{3}\right) \right) \cap L^{2}\left( \Omega ;L^{2}\left( 0,T;\mathcal {W }^{2,2}\left( \mathbb {T}^{2}\right) ^{3}\right) \right) \end{aligned}$$

for any $T>0$. In particular,

14 $$\begin{aligned} \mathbb {E}[\Vert a\Vert _{\tau ^R\wedge T,2,2}^2] <\infty \end{aligned}$$

for any $$R,T>0$$.

#### Proof

Immediate from Theorem 3.2 after observing that $a=a^R$ on $[0,\tau ^R]$ and $a^R \in \mathcal {M}_T$.

We are now ready to show the equality between the two stopping times $\mathcal {T}$ and ${\bar{\mathcal {T}}}$. $\square$

#### Proposition 3.6

Let $(a, \mathcal {T})$ be the maximal solution constructed in Proposition 3.4. Then

$$\begin{aligned} \mathbb {P}\left( \mathcal {T}= \hat{\mathcal {T}} \right) = 1. \end{aligned}$$

#### Proof

One can observe that $\hat{\tau }^M \le \tau ^M$ for all $M\ge 0$, and therefore $\hat{\mathcal {T}} \le \mathcal {T}$ $\mathbb {P}-a.s.$. We show that $\mathcal {T}\le \hat{\mathcal {T}}$ $\mathbb {P}-a.s.$. On the set $\{ \hat{\mathcal {T}} = \infty \}$ the inequality is trivially true, so we only need to show that

$$\begin{aligned} \mathbb {P}\left( \left\{ \omega \in \Omega : \hat{\mathcal {T}}(\omega ) < \infty , \mathcal {T}(\omega ) \le \hat{\mathcal {T}}(\omega )\right\} \right) =1. \end{aligned}$$

Note that

$$\begin{aligned} \begin{aligned} \left\{ \omega \in \Omega : \displaystyle \Vert a(\omega )\Vert _{\tau ^N,2,2}< \infty \right\}&= \displaystyle \bigcup _M \left\{ \omega \in \Omega : \Vert a(\omega )\Vert _{\tau ^N,2,2}< M\right\} \\&= \displaystyle \bigcup _M \left\{ \omega \in \Omega : \tau ^N(\omega ) < \hat{\tau }^M(\omega )\right\} \end{aligned} \end{aligned}$$

and

$$\begin{aligned} \begin{aligned} \displaystyle \bigcup _M \left\{ \omega \in \Omega : \tau ^N(\omega )< \hat{\tau }^M(\omega )\right\} \subset \left\{ \omega \in \Omega : \hat{\mathcal {T}}(\omega )<\infty ,\tau ^N(\omega ) < \hat{\mathcal {T}}(\omega ) \right\} . \end{aligned} \end{aligned}$$

From Corollary 3.5 we deduce that

$$\begin{aligned} \begin{aligned} \mathbb {P}\left( \{ \omega \in \Omega : \Vert a(\omega )\Vert _{\tau ^N\wedge T,2,2} < \infty \} \right) =1, \ \ \ \forall N\in \mathbb {N}, \ \ \ T>0. \end{aligned} \end{aligned}$$

and, since $\limsup _{t\rightarrow \hat{\mathcal {T}}} \Vert a(\omega )\Vert _{t,2,2}=\infty$ on the set $\{\hat{\mathcal {T}}<\infty \}$, we deduce that

$$\begin{aligned} \mathbb {P}\left( \left\{ \omega \in \Omega : \hat{\mathcal {T}}(\omega )<\infty , \tau ^N(\omega ) \wedge T< \hat{\mathcal {T}}(\omega )\right\} \right) =1, \ \ \ \forall N\in \mathbb {N}, \ \ \ T>0, \end{aligned}$$

therefore

$$\begin{aligned} \begin{aligned}&\mathbb {P}\left( \left\{ \omega \in \Omega : \hat{\mathcal {T}}(\omega )<\infty , \tau ^N(\omega )<\hat{\mathcal {T}}(\omega )\right\} \right) \\&= \mathbb {P}\left( \bigcap _{L>1}\left\{ \omega \in \Omega : \hat{\mathcal {T}}(\omega )<\infty , \tau ^N(\omega )\wedge L < \hat{\mathcal {T}}(\omega )\right\} \right) \\&= 1. \end{aligned} \end{aligned}$$

Then we have

$$\begin{aligned} \begin{aligned}&\mathbb {P}\left( \{\omega \in \Omega : \hat{\mathcal {T}}(\omega )<\infty , \mathcal {T}(\omega ) \le \hat{\mathcal {T}}(\omega )\}\right) \\&\ge \mathbb {P}\left( \displaystyle \bigcap _N \left\{ \omega \in \Omega : \hat{\mathcal {T}}(\omega )<\infty , \tau ^N(\omega ) < \hat{\mathcal {T}}(\omega )\right\} \right) \\&=1. \end{aligned} \end{aligned}$$

$$\square $$

### Pathwise Uniqueness for the Truncated SRSW System

Let $a^{R,1}=\left( v^{R,1}, h^{R,1}\right)$ and $a^{R,2}=\left( v^{R,2}, h^{R,2}\right)$ be two solutions of the truncated system starting from the initial conditions $a_0^1$, $a_0^2 \in \mathcal {W}^{1,2}\left( \mathbb {T}^2\right) ^3$, respectively. We denote the corresponding differences by $\bar{a}^R := a^{R,1} - a^{R,2}$, $\bar{v}^R:= v^{R,1}-v^{R,2}$, $\bar{h}^R:=h^{R,1}-h^{R,2}$. Also $\bar{u}^R:= u^{R,1}-u^{R,2}$, $\bar{p}^R:= p^{R,1}-p^{R,2}$. Assume that $\tau _M^{R,i}$ are the stopping times defined as

$$\begin{aligned} \tau _M^{R,i}:= \displaystyle \inf _t\left\{ t\ge 0, \Vert a^{R,i}\Vert _{t,2,2}\ge M \right\} . \end{aligned}$$

Define $$\bar{\tau }_M^R := \tau _M^{R,1}\wedge \tau _M^{R,2}$$.

#### Remark 3.7

We have $\displaystyle \lim _{M\rightarrow \infty }\tau _M^{R,i}=\infty \ \ \mathbb {P}-a.s.$. This is because

$$\begin{aligned} \mathbb {P}\left( \tau _M^{R,i}\le N\right) = \mathbb {P}\left( \Vert a^{R,i}\Vert _{N,2,2} \ge M\right) \le \frac{\mathbb {E}\left[ \Vert a^{R,i}\Vert _{N,2,2}^2\right] }{M^2} \end{aligned}$$

hence

$$\begin{aligned} \mathbb {P}\left( \displaystyle \lim _{M\rightarrow \infty }\tau _M^{R,i}\le N \right) \le \displaystyle \lim _{M\rightarrow \infty }\mathbb {P}\left( \tau _M^{R,i}\le N\right) = 0. \end{aligned}$$

Then

$$\begin{aligned} \begin{aligned} \mathbb {P}\left( \displaystyle \lim _{M\rightarrow \infty }\tau _M^{R,i}=\infty \right)&= 1 - \mathbb {P}\left( \displaystyle \lim _{M\rightarrow \infty }\tau _M^{R,i}< \infty \right) \ge 1 - \displaystyle \sum _N\mathbb {P}\left( \displaystyle \lim _{M\rightarrow \infty }\tau _M^{R,i} < N\right) = 1. \end{aligned} \end{aligned}$$

Consequently, also $$\displaystyle \lim _{M\rightarrow \infty }\bar{\tau }_M^R = \infty $$.

#### Theorem 3.8

Let $a^{R,1}$, $a^{R,2}$ be two solutions of the truncated SRSW system (9), which take values in the space $L^2\left( \Omega , C\left( [0,T],\mathcal {W}^{1,2}\left( \mathbb {T}^2\right) ^3\right) \right) \cap L^2\left( \Omega , L^2\left( 0,T;\mathcal {W}^{2,2}\left( \mathbb {T}^2\right) ^3\right) \right)$ and start from the initial conditions $a_0^1$, $a_0^2 \in \mathcal {W}^{1,2}\left( \mathbb {T}^2\right) ^3$, respectively. Then there exists a constant $C=C(M)$ such that

$$\begin{aligned} \mathbb {E}\left[ \Vert \bar{a}_{t\wedge \bar{\tau }_M^R}^R\Vert _{1,2}^2\right] \le Ce^{Ct}\Vert \bar{a}_0\Vert _{1,2}^2, \end{aligned}$$

where $\bar{a}^R := a^{R,1} - a^{R,2}$ and $\bar{\tau }_M^R := \tau _M^{R,1}\wedge \tau _M^{R,2}.$ In particular, following from Remark 3.7, the truncated SRSW system (9) has a unique solution in the space

$$\begin{aligned} L^2\left( \Omega , C\left( [0,T],\mathcal {W}^{1,2}\left( \mathbb {T}^2\right) \right) \right) \cap L^2\left( \Omega , L^2\left( 0,T;\mathcal {W}^{2,2}\left( \mathbb {T}^2\right) \right) \right) . \end{aligned}$$

#### Proof

We show that

15 $$\begin{aligned} \begin{aligned} d\Vert \bar{a}_t^R\Vert _{1,2}^2 \le C(\epsilon ,R)\Vert Z_t\Vert \Vert \bar{a}_t^R\Vert _{1,2}^2 dt + dB_t \end{aligned} \end{aligned}$$

where $$\epsilon > 0$$,

$$\begin{aligned} \Vert Z_t\Vert := C\left( \Vert a_t^{R,1}\Vert _{1,2}^4 + \Vert a_t^{R,2}\Vert _{1,2}^4\right) , \end{aligned}$$

and $dB_t$ is a local martingale given by

16 $$\begin{aligned} dB_t := - 2\displaystyle \sum _{i=1}^{\infty }\left( \langle \bar{v}_t^R, \mathcal {G}_i\bar{v}_t^R\rangle + \langle \bar{h}_t^R, \mathcal {L}_i \bar{h}_t^R\rangle + \langle \Delta \bar{v}_t^R, \mathcal {G}_i\bar{v}_t^R\rangle + \langle \Delta \bar{h}_t^R, \mathcal {L}_i\bar{h}_t^R \rangle \right) dW_t^i. \end{aligned}$$

Then

$$\begin{aligned} \begin{aligned} \mathbb {E}\left[ e^{-C\displaystyle \int _0^{t\wedge \bar{\tau }_M^R}\Vert Z_s\Vert ds} \Vert \bar{a}_{t\wedge \bar{\tau }_M^R}^R\Vert _{1,2}^2\right]&\le \Vert \bar{a}_0\Vert _{1,2}^2 + \mathbb {E}\left[ \displaystyle \int _0^{t\wedge \bar{\tau }_M^R}e^{-C\displaystyle \int _0^{s\wedge \bar{\tau }_M^R}\Vert Z_r\Vert dr}dB_{s}\right] \end{aligned} \end{aligned}$$

that is

$$\begin{aligned} \begin{aligned} \mathbb {E}\left[ \Vert \bar{a}_{t\wedge \bar{\tau }_M^R}^R\Vert _{1,2}^2\right]&\le e^{CM^4t}\Vert \bar{a}_0\Vert _{1,2}^2 \end{aligned} \end{aligned}$$

since the stopped process $B_{t\wedge \bar{\tau }_M^R}$ is a martingale. By choosing two solutions of the truncated SRSW system (9) which start from the same initial conditions, we deduce that $\mathbb {P}\left( a_s^{R,1} = a_s^{R,2}, \forall s\in [0, \bar{\tau }_M^R]\right) =1$ for any $M>0$, that is the two solutions are indistinguishable. Since $\displaystyle \lim _{M\rightarrow \infty }\bar{\tau }_M^R = \infty$ we deduce that the solution is unique globally. We will now prove that (15) holds, using Lemma 6.1. We can write

17a $$\begin{aligned} d\bar{v}_t^R&= \left( -Q_{\bar{v}^R} - fk\times \bar{v}_t^R + \nu \Delta \bar{v}_t^R - g\nabla \bar{p}_t^R + \frac{1}{2}\displaystyle \sum _{i=1}^{\infty }(\mathcal {L}_i+\mathcal {A}_i)^2\bar{v}_t^R \right) dt \nonumber \\&\quad - \displaystyle \sum _{i=1}^{\infty }(\mathcal {L}_i+\mathcal {A}_i)\bar{v}_t^RdW_t^i \end{aligned}$$

17b $$\begin{aligned}&\quad d\bar{h}_t^R = \left( - Q_{\bar{h}^R} + \eta \Delta \bar{h}_t^R+ \frac{1}{2}\displaystyle \sum _{i=1}^{\infty }\mathcal {L}_i^2\bar{h}_t^R \right) dt - \displaystyle \sum _{i=1}^{\infty }\mathcal {L}_i\bar{h}_t^R dW_t^i . \end{aligned}$$

where

$$\begin{aligned}{} & {} Q_{\bar{v}^R}:=f_R\left( a_R^1\right) u_t^{R,1}\cdot \nabla v_t^{R,1} - f_R\left( a_R^2\right) v_t^{R,2}\cdot \nabla u_t^{R,2} \\{} & {} \quad Q_{\bar{h}^R} := f_R\left( a_R^1\right) \nabla \cdot \left( h_t^{R,1}u_t^{R,1}\right) - f_R\left( a_R^2\right) \nabla \cdot \left( h_t^{R,2}u_t^{R,2}\right) . \end{aligned}$$

By the Itô formula

$$\begin{aligned} \begin{aligned}&d\Vert \bar{a}_t^R\Vert _{1,2}^2 + 2\gamma \Vert \bar{a} _t^R\Vert _{2,2}^2dt \\&\le 2\left( \langle \Delta \bar{v}_t^R-\bar{v}_t^R, Q_{\bar{v}_t^R}\rangle + \langle \Delta \bar{h}_t^R-\bar{h}_t^R, Q_{\bar{h}_t^R}\rangle +\langle \Delta \bar{v}_t^R - \bar{v}_t^R ,fk\times \bar{v}_t^R + g\nabla \bar{p}_t^R\rangle \right) dt \\&\quad + \displaystyle \sum _{i=1}^{\infty } \left( \langle (\mathcal {L}_i+\mathcal {A}_i)\bar{v}_t^R, (\mathcal {L}_i+\mathcal {A}_i) \bar{v}_t^R \rangle _{1,2} + \langle \mathcal {L}_i\bar{h}_t^R, \mathcal {L }_i\bar{h}_t^R\rangle _{1,2} \right) dt \\&\quad - \displaystyle \sum _{i=1}^{\infty } \left( \langle \Delta \bar{v}_t^R,(\mathcal {L}_i+\mathcal {A}_i)^2\bar{v}_t^R\rangle + \langle \Delta \bar{h}_t^R, \mathcal {L}_i^2\bar{h} _t^R\rangle \right) dt \\&\quad + 2\displaystyle \sum _{i=1}^{\infty }\left( \langle \Delta \bar{v}_t^R-\bar{v}_t^R, (\mathcal {L}_i+\mathcal {A}_i) \bar{v}_t^R\rangle + \langle \Delta \bar{h}_t^R - \bar{h}_t^R, \mathcal {L}_i \bar{h}_t^R\rangle \right) dW_t^i. \end{aligned} \end{aligned}$$

All the terms which do not contain a stochastic integral are controlled as functions of $C(\zeta ,R)\Vert Z\Vert \Vert \bar{a}\Vert _{1,2}^2 + \zeta \Vert \bar{a}\Vert _{2,2}^2$ using Lemma 6.1, Lemma 6.2, and assumption (6) respectively. We choose $\zeta < \gamma$ such that all the terms which are controlled by $\zeta \Vert \bar{a}\Vert _{2,2}^2$ on the right hand side cancel out the term $2\gamma \Vert \bar{a}\Vert _{2,2}^2$ on the left hand side. Then (15) holds as requested and therefore the two solutions are indistinguishable as processes with paths in $L^2\left( \Omega , C\left( [0,T],\mathcal {W}^{1,2}\left( \mathbb {T}^2\right) ^3\right) \right) \cap L^2\left( \Omega , L^2\left( 0,T;\mathcal {W}^{2,2}\left( \mathbb {T}^2\right) ^3\right) \right)$. $\square$

#### Remark 3.9

From Proposition 3.6, we deduce that $\displaystyle \lim _{M\rightarrow \infty }\tau _M^{i}=\tilde{\mathcal {T}}^i\ \ \mathbb {P}-a.s.$, for $i=1,2$. Consequently, also $\displaystyle \lim _{M\rightarrow \infty }\bar{\tau }_M = \tilde{\mathcal {T}}^1 \wedge \tilde{\mathcal {T}}^2$, where $\bar{\tau }_M := \tau _M^1 \wedge \tau _M^2$. Moreover, $a^i=a^{M,i}$ on $[0,\tau ^{M,i}]$ for $i=1,2$ and arbitrary $M>0$, therefore $\tau ^i_M=\tau ^{M,i}_M$.

#### Corollary 3.10

Let $\left( a^1, \mathcal {T}^1\right)$ and $\left( a^2, \mathcal {T}^2\right)$ be two maximal solutions of the original system, starting from $a_0^1, a_0^2 \in \mathcal {W}^{1,2}\left( \mathbb {T}^2\right) ^3$, respectively. Then there is a constant $C=C(M)$ such that

$$\begin{aligned} \mathbb {E}\left[ \Vert \bar{a}_{t\wedge \bar{\tau }_M}\Vert _{1,2}^2\right] \le Ce^{Ct}\Vert a_0^1-a_0^2\Vert _{1,2}^2. \end{aligned}$$

#### Proof

From Remark 3.9 and Theorem 3.8 we deduce that

$$\begin{aligned} \mathbb {E}\left[ \Vert \bar{a}_{t\wedge \bar{\tau }_M}\Vert _{1,2}^2\right] = \mathbb {E}\left[ \Vert \bar{a}_{t\wedge \bar{\tau }_M^M}\Vert _{1,2}^2\right] \le Ce^{Ct}\Vert a_0^1-a_0^2\Vert _{1,2}^2. \end{aligned}$$

$$\square $$

#### Remark 3.11

Note that $\displaystyle \lim _{M\rightarrow \infty }\tau _M^i = \tau ^i$ (the maximal time of existence) so the continuity covers the common interval of existence.

### Global Existence for the Truncated SRSW System

#### Proposition 3.12

The truncated SRSW system (9) admits a global solution $a^R=\left( v^R, h^R\right)$ such that $a^R_{[0,T]} \in \mathcal {M}_T$ for any $T\ge 0$. In other words

$$\begin{aligned} a^R_{[0,T]} \in L^2\left( \Omega , C\left( [0,T],\mathcal {W}^{1,2}\left( \mathbb {T}^2\right) \right) \right) \cap L^2\left( \Omega , L^2\left( 0,T;\mathcal {W}^{2,2}\left( \mathbb {T}^2\right) ^3\right) \right) \end{aligned}$$

for any $T>0$. Moreover

$$\begin{aligned} a^R_{[0,T]} \in L^p\left( \Omega , \mathcal {W}^{\alpha ,p}([0,T],L^{2}(\mathbb {T}^2)^3)\right) \end{aligned}$$

for any $p\in (2, \infty )$ and $\alpha \in \left[ 0, \frac{1}{2}\right)$ such that $p\alpha > 1$ and

$$\begin{aligned} a^R_{[0,T]} \in L^p\left( \Omega , C([0,T],\mathcal {W}^{1,2}(\mathbb {T}^2))\right) \end{aligned}$$

for any $$T>0$$.

In the following we will omit the dependence of the truncated system $a^R=\left( v^R,h^R\right)$ on *R* and simply use the notation $a=(v,h)$ to denote it. The strategy for proving that the truncated system (3.2) has a solution is to construct an approximating system of processes that will converge in distribution to a solution of (3.2). This justifies the existence of a weak solution. Together with the pathwise uniqueness of the solution of this equation, we then deduce that strong/pathwise existence holds.

Recall that $\left( v_{0},h_{0}\right) \in \mathcal {W}^{1,2}\left( \mathbb {T} ^{2}\right) ^2\times \mathcal {W}^{1,2}\left( \mathbb {T}^{2}\right)$. We construct the sequence $\left( v^{n},h^{n}\right) _{n\ge 0}$ with $v_{t}^{0}:=u_{0}^{0},$ $h_{t}^{0}:=h_{0}^{0}$ , and for $n\ge 1,$ we define $(v^{n},h^{n})_{n\ge 0}$ as the solution of the *linear* SPDE

$$\begin{aligned} \begin{aligned}&dv_{t}^{n}=\nu \Delta v_{t}^{n}dt+P_{t}^{n-1,n}\left( v_{t}^{n}\right) dt-\sum _{i=1}^{\infty }\left( \mathcal {L}_{i}+ \mathcal {A}_{i}\right) v_{t}^{n}dW_{t}^{i,n} \\&dh_{t}^{n}=\delta \Delta h_{t}^{n}dt+Q_{t}^{n-1,n}\left( h_{t}^{n}\right) dt-\sum _{i=1}^{\infty }\nabla \cdot (\xi _{i}h_{t}^{n})dW_{t}^{i,n}, \end{aligned} \end{aligned}$$

where $$ P_{t}^{n-1,n}(v_{t}^{n})$$ and $$Q_{t}^{n-1,n}(v_{t}^{n})$$ are defined, respectively, as follows (for $$t\ge 0$$):

$$\begin{aligned} \begin{aligned} P_{t}^{n-1,n}\left( v_{t}^{n}\right)&:=-f_{R}\left( a_{t}^{n-1}\right) \mathcal {L} _{u_{t}^{n-1}}v_{t}^{n-1}-f\hat{z}\times u_{t}^{n}-\nabla p_{t}^{n}+\frac{1}{ 2}\sum _{i=1}^{\infty }\left( \mathcal {L}_{i}+\mathcal {A}_{i}\right) ^{2}v_{t}^{n} \\&Q_{t}^{n-1,n}\left( h_{t}^{n}\right) :=-f_{R}\left( a_{t}^{n-1}\right) \left( \nabla \cdot \left( h_{t}^{n-1}u_{t}^{n-1}\right) \right) +\frac{1}{2}\sum _{i=1}^{\infty } \mathcal {L}_{i}^{2}h_{t}^{n} \end{aligned} \end{aligned}$$

#### Theorem 3.13

The approximating system admits a unique global solution in the space

$$\begin{aligned} \left( v^{n},h^{n}\right) \in L^{2}\left( \Omega ;C\left( [0,T];\mathcal {W}^{1,2}\left( \mathbb {T}^{2}\right) ^{3}\right) \right) \cap L^{2}\left( \Omega ;L^{2}\left( 0,T; \mathcal {W}^{2,2}\left( \mathbb {T}^{2}\right) ^{3}\right) \right) \end{aligned}$$

and for any $p\ge 2$ there exists a constant $\mathcal {B}_{3}(T,R)$ independent of *n* such that

18 $$\begin{aligned} \mathbb {E}\left[ \left\| \left( v^{n},h^{n}\right) \right\| _{T,2,2}^{p}\right] \le \mathcal {B}_{3}(T,R). \end{aligned}$$

Moreover $\left( v^{n},h^{n}\right) \in L^{p}\left( \Omega ;\mathcal {W}^{\alpha ,p}\left( [0,T], L^{2}\left( \mathbb {T}^{2}\right) ^{3}\right) \right)$ with $p \in (2,\infty ),\alpha \in [0,\frac{1}{2})$ such that $p\alpha > 1$and there exists a constant $\mathcal {B}_{4}(p,\alpha ,T,R)$ independent of *n* such that

19 $$\begin{aligned} \mathbb {E}\left[ \left\| \left( v^{n},h^{n}\right) \right\| _{\mathcal {W}^{\alpha , p}\left( [0,T],L^{2}\left( \mathbb {T}^{2}\right) ^{3}\right) }^p \right] \le \mathcal {B}_{4}(p,T, R). \end{aligned}$$

The proof of this theorem is provided in Sect. 5 below.

#### Proposition 3.14

The family of probability distributions of the solutions $\left( v^{n},h^{n}\right) _{n}$ is relatively compact in the space of probability measures over $L^{2}\left( \Omega ;C\left( [0,T];L^{2}\left( \mathbb {T} ^{2}\right) ^{3}\right) \right)$ for any $T\ge 0$.

Proposition 3.14 is proven in Sect. 5.

#### Proof of Proposition 3.12

It is in the proof of this proposition that we see the additional difficulties encountered for stochastic models as compared to the deterministic models. Let us explain why this is the case. Recall that Proposition 3.14 tells us that the family of *probability distributions* of the approximate solutions $$ (v^n,h^n)_n$$ is relatively compact over $$L^{2}\left( \Omega ;C\left( [0,T];L^{2}(\mathbb {T} ^{2})^{3}\right) \right) $$ for any $$T\ge 0$$. This does not mean that the processes themselves are relatively compact. Therefore, in contrast to the deterministic case, we cannot extract a subsequence from $$(v^n,h^n)_n$$ that will converge pathwise. We can only extract a subsequence $$(v^{n_j},h^{n_j})$$ that will converge in distribution over $$L^{2}\left( \Omega ;C\left( [0,T];L^{2}(\mathbb {T}^{2})^{3}\right) \right) $$ for any $$T\ge 0$$. We can then find a *different* probability space $$(\tilde{\Omega },\tilde{{\mathcal {F}}}, \tilde{\mathbb {P}})$$ on which we can build copies of $$(v^{n_j},h^{n_j})$$ with the same distributions as the original ones that converge in $$L^{2}\left( \tilde{\Omega };C\left( [0,T];L^{2}(\mathbb {T} ^{2})^{3}\right) \right) $$ and, possibly by using a further subsequence, we can also assume that the convergence is pathwise. This is done by means of a classical probabilistic result called the Skorokhod representation theorem, see for example [1] Section 6, pp. 70.

Further complications need to be sorted: It is not enough to transfer just the processes $(v^{n_j},h^{n_j})$. The driving Brownian motions $(W_i)_{i=1}^{\infty }$ will need to be mirrored in the new space $(\tilde{\Omega },\tilde{{\mathcal {F}}}, \tilde{\mathbb {P}})$ as the "mirroring process" is done for each individual term of the subsequence. We end up with a set of Brownian motions that are different for each element of the sequence, even if we start with a subsequence that is driven by the same set of Brownian motions (therefore we do not have to drive the original sequence with the same set of Brownian motions as only the convergence of the probability distributions of $(v^{n_j},h^{n_j})$ will matter in the first place. The next step will be to show that, on the new probability space $(\tilde{\Omega },\tilde{{\mathcal {F}}}, \tilde{\mathbb {P}})$, the mirror sequence converges to a solution of the truncated equation. Since the convergence of the mirror sequence holds only in $L^{2}\left( \tilde{\Omega };C\left( [0,T];L^{2}(\mathbb {T} ^{2})^{3}\right) \right)$, we will need to resort to the weak (in probabilistic sense) version of the equation satisfied by the mirror image of $(v^{n_j},h^{n_j})$. Let us ignore the choice of the subsequence and denote the mirror sequence by $((\tilde{v}^n, \tilde{h}^n), (\tilde{W} ^{i,n})_i)_{n=1}^{\infty }$. Note that we added the corresponding set Brownian motions for each element of the sequence in the light of the discussion from above. Then, for any test function $\varphi \in W^{3,2}( \mathbb {T}^2)$, we can write

20 $$\begin{aligned} \langle \tilde{v}_t^n,\varphi \rangle&= \langle \tilde{v}_0^n, \varphi \rangle + \nu \displaystyle \int _0^t\langle \tilde{v}_s^n, \Delta \varphi \rangle ds - \displaystyle \int _0^t f_R(\tilde{a}_s^{n-1})\langle \tilde{v}_s^{n-1}, \mathcal {L}_{\tilde{u}_s^{n-1}}^{\star } \varphi \rangle ds - \displaystyle \int _0^t \langle f\hat{z} \times \tilde{u}_s^n, \varphi \rangle ds \nonumber \\&\quad + \displaystyle \int _0^t \langle \tilde{p}_s^{n}, \nabla \varphi \rangle ds + \frac{1}{2}\displaystyle \sum _{i=1}^{\infty }\displaystyle \int _0^t \langle \tilde{v}_s^n, (\mathcal {L}_i^{\star } + \mathcal {A} _i^{\star })^2\varphi \rangle ds - \displaystyle \sum _{i=1}^{\infty }\displaystyle \int _0^t\langle \tilde{v} _s^n,(\mathcal {L}_i^{\star } + \mathcal {A}_i^{\star }) \varphi \rangle d{\tilde{W}}_s^{i,n} \end{aligned}$$

21 $$\begin{aligned} \langle \tilde{h}_t^n, \varphi \rangle&= \langle \tilde{h}_0^n, \varphi \rangle + \eta \displaystyle \int _0^t \langle \tilde{h}_s^n, \Delta \varphi \rangle ds + \displaystyle \int _0^t f_R(\tilde{a}_s^{n-1}) \langle \nabla \varphi , \tilde{h}_s^{n-1} \tilde{u}_s^{n-1} \rangle ds \nonumber \\&\quad + \frac{1}{2} \displaystyle \sum _{i=1}^{\infty }\displaystyle \int _0^t\langle \tilde{h}_s^n, ( \mathcal {L}_i^{\star })^2 \varphi \rangle ds - \displaystyle \sum _{i=1}^{\infty } \displaystyle \int _0^t \langle \tilde{h}_s^n, \mathcal {L}_i^{\star }\varphi \rangle d{\tilde{W}}_s^{i,n}. \end{aligned}$$

The next step would be to pass to the limit in (20) and (21) and show that each term converges to the corresponding term in the equation satisfied by the truncated system. The convergence of the stochastic integrals in (20) and (21) poses an additional difficulty. The reason is that, even though we know that both the integrands and the integrators (the driving Brownian motions) converge, that does not necessarily imply that the corresponding integrals converge. To circumvent this hurdle we make use of Theorem 4.2 in [21] which states that if the integrands and the integrators converge in distribution (in the original space), then the stochastic integrals converge in distribution too. Then, via the Skorokhod representation theorem, we find a mirror probability space $(\tilde{\Omega },\tilde{{\mathcal {F}}}, \tilde{\mathbb {P}})$ where, *by construction*, not only $((\tilde{v}^n, \tilde{h}^n), (\tilde{W}^{i,n})_i)_{n=1}^{\infty }$ converge, but also the corresponding stochastic integrals. We proceed with the construction as follows:

We choose $\left\{ \varphi _{k}\right\} _{k}$ to be a countable dense set of $\mathcal {W}^{2,2}(\mathbb {T}^{2})$. By Proposition 3.14 and Theorem 4.2 in [21] we can deduce that the processes

$$\begin{aligned} \begin{aligned}\{v^{n},h^{n},&\int _{{0}}^{\cdot }\left\langle v_{1}^{n},\left( \mathcal {L}_{i}+\mathcal {A}_{i}\right) ^{*}\varphi _{k_{1}}\right\rangle _{L^{2}(\mathbb {T}^{2})}dW_{s}^{i_{1},n}, \int _{{0}}^{\cdot }\left\langle v_{2}^{n},\left( \mathcal {L}_{i}+\mathcal {A}_{i}\right) ^{*}\varphi _{k_{2}}\right\rangle _{L^{2}(\mathbb {T}^{2})}dW_{s}^{i_{2},n}, \\ {}&\int _{{0}}^{\cdot }\left\langle h^{n},\mathcal {L}_{i}\varphi _{k}\right\rangle _{L^{2}(\mathbb {T}^{2})}dW_{s}^{i_{3},n} \text {,~~}i_{1},i_{2},i_{3},k_{1},k_{2},k_{3}=1,...\infty ,\}_{n=1}^{\infty } \end{aligned} \end{aligned}$$

converge in distribution (possibly by extracting a subsequence of the original sequence and re-indexing it). We apply next the Skorokhod representation theorem to this (enlarged) sequence and find a probability space $(\tilde{\Omega },\tilde{{\mathcal {F}}}, \tilde{\mathbb {P}})$ on which we can find processes

$$\begin{aligned} \begin{aligned}\{{\tilde{v}}^{n},{\tilde{h}}^{n},&\int _{{0}}^{\cdot }\left\langle {\tilde{v}}_{1}^{n},\left( \mathcal {L}_{i}+\mathcal {A}_{i}\right) ^{*}\varphi _{k_{1}}\right\rangle _{L^{2}(\mathbb {T}^{2})}d{\tilde{W}}_{s}^{i_{1},n}, \int _{{0}}^{\cdot }\left\langle {\tilde{v}}_{2}^{n},\left( \mathcal {L}_{i}+\mathcal {A}_{i}\right) ^{*}\varphi _{k_{2}}\right\rangle _{L^{2}(\mathbb {T}^{2})}d \tilde{W}_{s}^{i_{2},n}, \\&\int _{{0}}^{\cdot }\left\langle {\tilde{h}}^{n},\mathcal {L}_{i}\varphi _{k}\right\rangle _{L^{2}(\mathbb {T}^{2})}d{\tilde{W}}_{s}^{i_{3},n} \text {,~~}i_{1},i_{2},i_{3},k_{1},k_{2},k_{3}=1,...\infty ,\}_{n=1}^{\infty } \end{aligned} \end{aligned}$$

with the same probability distributions as the corresponding elements of the original sequence and that converge to

$$\begin{aligned} \begin{aligned}({\tilde{v}},{\tilde{h}},&\int _{{0}}^{\cdot }\left\langle {\tilde{v}}_{1},\left( \mathcal {L}_{i}+\mathcal {A}_{i}\right) ^{*}\varphi _{k_{1}}\right\rangle _{L^{2}(\mathbb {T}^{2})}d\tilde{W}_{s}^{i_{1}}, \int _{{0}}^{\cdot }\left\langle {\tilde{v}}_{2},\left( \mathcal {L}_{i}+\mathcal {A}_{i}\right) ^{*}\varphi _{k_{2}}\right\rangle _{L^{2}(\mathbb {T}^{2})}d \tilde{W}_{s}^{i_{2}}, \\&\int _{{0}}^{\cdot }\left\langle {\tilde{h}},\mathcal {L}_{i}\varphi _{k}\right\rangle _{L^{2}(\mathbb {T}^{2})}d{\tilde{W}}_{s}^{i_{3}} \text {,~~}i_{1},i_{2},i_{3},k_{1},k_{2},k_{3}=1,...\infty ) \end{aligned} \end{aligned}$$

in the corresponding product spaces as well as pathwise (possibly by extracting a suitable subsequence).

It follows that all the estimates established for $(v^n,h^n)$ are also true for $(\tilde{v}^n,\tilde{h}^n)$. Thus, there exists a constant $\tilde{\mathcal {B}}_{3}(T,R)$ such that

22 $$\begin{aligned} \tilde{\mathbb {E}}\left[ \left| \left| ({\tilde{v}}^{n},{\tilde{h}}^{n})\right| \right| _{T,2,2}^{2}\right] \le \tilde{\mathcal {B}}_{3}(T,R), \end{aligned}$$

which ensures that the corresponding time integrals of the terms involved are uniformly bounded in $L^2(\tilde{\mathbb {P}})$, and, by Fatou’s lemma, also that

23 $$\begin{aligned} \tilde{\mathbb {E}}\left[ \left| \left| ({\tilde{v}},{\tilde{h}})\right| \right| _{T,2,2}^{2}\right] \le \tilde{\mathcal {B}}_{3}(T,R), \end{aligned}$$

Similarly, we also have that $({\tilde{v}}^{n},{\tilde{h}}^{n})\in L^{p}\left( \tilde{\Omega };\mathcal {W}^{\alpha ,p}\left( [0,T], L^{2}(\mathbb {T}^{2})^{3}\right) \right)$ with $p \in (2,\infty ),\alpha \in [0,\frac{1}{2})$ such that $p\alpha > 1$ and there exists a constant $\mathcal {B}_{4}(p,\alpha ,T,R)$ independent of *n* such that

24 $$\begin{aligned} \mathbb {{\tilde{E}}}\left[ \left| \left| ({\tilde{v}}^{n},{\tilde{h}}^{n})\right| \right| _{\mathcal {W}^{\alpha , p}\left( [0,T],L^{2}(\mathbb {T}^{2})^{3}\right) } \right] \le \mathcal {B}_{4}(p,T, R). \end{aligned}$$

with the same control applying to the limit process $({\tilde{v}},{\tilde{h}})\in L^{p}\left( \tilde{\Omega };\mathcal {W}^{\alpha ,p}\left( [0,T], L^{2}(\mathbb {T}^{2})^{3}\right) \right)$. We pass to the limit in all the terms in (20) and (21). The stochastic terms converge by construction, therefore we only need to concentrate on the deterministic terms. Of these, the convergence of the linear terms is straightforward and relies on the convergence of $({\tilde{v}}^{n},{\tilde{h}}^{n})$ in $L^{2}\left( \tilde{\Omega };C(\left( [0,T];L^{2}(\mathbb {T}^{2})^{3}\right) \right)$. We detail next the convergence of the nonlinear terms. For the velocity equation we show that

$$\begin{aligned} \displaystyle \int _0^t \langle f_R(a_s^{n-1})\mathcal {L}_{\tilde{u}_s^{n-1}}\tilde{v} _s^{n-1} - f_R(a_s^R)\mathcal {L}_{\tilde{u}_s^{R}}\tilde{v}_s^R , \varphi \rangle ds \xrightarrow [n\rightarrow \infty ]{} 0 \ \ \hbox {in} \ L^2(\tilde{\mathbb {P}}). \end{aligned}$$

One can split this difference as follows

$$\begin{aligned} \begin{aligned} |\langle f_R(a_s^{n-1})\mathcal {L}_{\tilde{u}_s^{n-1}}\tilde{v}_s^{n-1} - f_R(a_s^R)\mathcal {L}_{\tilde{u }_s^{R}}\tilde{v}_s^R , \varphi \rangle |&\le f_R(a_s^R)|\langle (\tilde{u}_s^{n-1} - \tilde{u}_s^R) \cdot \nabla \tilde{v}_s^{n-1}, \varphi \rangle | \\&\quad + f_R(a_s^R)|\langle \tilde{u} _s^R \cdot \nabla (\tilde{v}_s^{n-1}-\tilde{v}_s^R), \varphi \rangle | \\&\quad + |f_R(a_s^{n-1})-f_R(a_s^R)||\langle \tilde{u}_s^{n-1} \cdot \nabla \tilde{v}_s^{n-1}, \varphi \rangle |. \end{aligned} \end{aligned}$$

For the first term we have

$$\begin{aligned} \begin{aligned}&\mathbb {E}\left[ \displaystyle \int _0^t f_R(a_s^R)|\langle (\tilde{u}_s^{n-1} - \tilde{u}_s^R) \cdot \nabla \tilde{v}_s^{n-1}, \varphi \rangle |ds \right] \\&\le C(\Vert \varphi \Vert _{\infty })\mathbb {E}\left[ \displaystyle \sup _{s\in [0,t]}\Vert \tilde{u}_s^{n-1} - \tilde{u}_s^R\Vert _2\displaystyle \int _0^t\Vert \nabla \tilde{v}_s^{n-1}\Vert _2 ds\right] \\&\le C(\Vert \varphi \Vert _{2,2})\sqrt{\mathbb {E}\left[ \displaystyle \sup _{s\in [0,t]}\Vert \tilde{u}_s^{n-1} - \tilde{u}_s^R\Vert _2^2\right] \mathbb {E}\left[ \displaystyle \int _0^t\Vert \nabla \tilde{v}_s^{n-1}\Vert _2^2 ds \right] } \\&\le C(t, \Vert \varphi \Vert _{2,2})\sqrt{\mathbb {E}\left[ \displaystyle \sup _{s\in [0,t]}\Vert \tilde{u}_s^{n-1} - \tilde{u}_s^R\Vert _2^2\right] \mathbb {E}\left[ \displaystyle \sup _{s\in [0,t]}\Vert \tilde{v}_s^{n-1}\Vert _{1,2}^2 \right] } \\&\le C(t, \Vert \varphi \Vert _{2,2})\mathbb {E}\left[ \displaystyle \sup _{s\in [0,t]}\Vert \tilde{u}_s^{n-1} - \tilde{u}_s^R\Vert _2^2\right] ^{1/2} \end{aligned} \end{aligned}$$

and the term on the right hand side converges to 0 in $L^2(\tilde{\mathbb {P}})$ and all other terms are controlled uniformly in *n*. For the second term,

$$\begin{aligned} \begin{aligned}&\mathbb {E}\left[ \displaystyle \int _0^t f_R(a_s^R)|\langle \tilde{u} _s^R \cdot \nabla (\tilde{v}_s^{n-1}-\tilde{v}_s^R), \varphi \rangle |ds\right] = \mathbb {E}\left[ \displaystyle \int _0^t f_R(a_s^R)|\langle \tilde{u} _s^R \cdot (\tilde{v}_s^{n-1}-\tilde{v}_s^R), \nabla \varphi \rangle |ds\right] \\&\quad + \mathbb {E}\left[ \displaystyle \int _0^t f_R(a_s^R)|\langle (\nabla \cdot \tilde{u} _s^R )\cdot (\tilde{v}_s^{n-1}-\tilde{v}_s^R), \varphi \rangle |ds\right] \\&\le \mathbb {E}\left[ \displaystyle \sup _{s\in [0,t]}\Vert \tilde{v}_s^{n-1}-\tilde{v}_s^R\Vert _2\displaystyle \int _0^tf_R(a_s^R)\Vert \tilde{u}_s^R \cdot \nabla \varphi \Vert _2ds \right] \\ {}&\quad +C(\Vert \varphi \Vert _{\infty }) \mathbb {E}\left[ \displaystyle \sup _{s\in [0,t]}\Vert \tilde{v}_s^{n-1}-\tilde{v}_s^R\Vert _2 \displaystyle \int _0^tf_R(a_s^R)\Vert \nabla \cdot \tilde{u}_s^R\Vert _2ds\right] \\&\le C \sqrt{\mathbb {E}\left[ \displaystyle \sup _{s\in [0,t]}\Vert \tilde{v}_s^{n-1}-\tilde{v}_s^R\Vert _2^2\right] \mathbb {E}\left[ \displaystyle \int _0^t f_R(a_s^R)^2\Vert \tilde{u}_s^R\Vert _{1,2}^2\Vert \nabla \varphi \Vert _{1,2}^2\right] } \\&\quad +C(\Vert \varphi \Vert _{2,2}) \sqrt{\mathbb {E}\left[ \displaystyle \sup _{s\in [0,t]}\Vert \tilde{v}_s^{n-1}-\tilde{v}_s^R\Vert _2^2\right] \mathbb {E}\left[ \displaystyle \int _0^tf_R(a_s^R)^2\Vert \tilde{u}_s^R\Vert _{1,2}^2ds\right] } \\&\le C(t,\Vert \varphi \Vert _{2,2}, R)\mathbb {E}\left[ \displaystyle \sup _{s\in [0,t]}\Vert \tilde{v}_s^{n-1}-\tilde{v}_s^R\Vert _2^2\right] ^{1/2} \ \ \xrightarrow [n\rightarrow \infty ]{}0. \end{aligned} \end{aligned}$$

For the third term,

$$\begin{aligned} \begin{aligned}&\mathbb {E}\left[ \displaystyle \int _0^t|f_R(a_s^{n-1})-f_R(a_s^R)||\langle \tilde{u}_s^{n-1} \cdot \nabla \tilde{v}_s^{n-1}, \varphi \rangle |ds\right] \\&\quad \le C(\Vert \varphi \Vert _{\infty }) \sqrt{\mathbb {E}\left[ \displaystyle \int _0^t|f_R(a_s^{n-1})-f_R(a_s^R)|^2ds\right] \mathbb {E}\left[ \displaystyle \int _0^t\Vert \tilde{u}_s^{n-1} \cdot \nabla \tilde{v}_s^{n-1}\Vert _2^2ds\right] } \\&\quad \le C(t,\Vert \varphi \Vert _{2,2})\sqrt{\mathbb {E}\left[ \displaystyle \sup _{s\in [0,t]}\Vert a_s^{n-1}-a_s^R\Vert _2\displaystyle \int _0^t\Vert a_s^{n-1}-a_s^R\Vert _{2,2}ds\right] \mathbb {E}\left[ \displaystyle \sup _{s\in [0,t]}\Vert a_s^{n-1}\Vert _{1,2}^2\right] } \\&\quad \le C(t,\Vert \varphi \Vert _{2,2})\mathbb {E}\left[ \displaystyle \sup _{s\in [0,t]}\Vert a_s^{n-1}-a_s^R\Vert _2^2\right] ^{1/4}\mathbb {E}\left[ \displaystyle \int _0^t(\Vert a_s^{n-1}\Vert _{2,2}^2 + \Vert a_s^R\Vert _{2,2}^2) ds\right] ^{1/4} \\&\quad \le \tilde{C}(t,\Vert \varphi \Vert _{2,2})\mathbb {E}\left[ \displaystyle \sup _{s\in [0,t]}\Vert a_s^{n-1}-a_s^R\Vert _2^2\right] ^{1/4} \ \ \xrightarrow [n\rightarrow \infty ]{}0. \end{aligned} \end{aligned}$$

Note that $\mathbb {E}\left[ \displaystyle \int _0^t\Vert a_s^R\Vert _{2,2}^2 ds\right] < \infty$ by a direct application of the Fatou lemma. With similar arguments, the nonlinear term in the height equation (21) converges as requested:

$$\begin{aligned}\displaystyle \int _0^t \langle f_R(a_s^{n-1}) \nabla \cdot (\tilde{h}_s^{n-1}\tilde{u} _s^{n-1}) - f_R(a_s^R)\nabla \cdot (\tilde{h}_s^{R}\tilde{u}_s^R), \varphi \rangle \rangle ds \xrightarrow [n\rightarrow \infty ]{} 0 \ \ \ \hbox {in} \ \ L^2(\tilde{\mathbb {P}}). \end{aligned}$$

We have constructed a weak (in PDE sense) solution of the SRSW system, as we have chosen the set of test functions $(\varphi _{k})_{k}$ to be a countable dense set of $\mathcal {W}^{2,2}(\mathbb {T}^{2})$. Since $({\tilde{v}}, {\tilde{h}})$ has the right amount of smoothness, this weak solution is also strong (in PDE sense). However, $({\tilde{v}}, {\tilde{h}})$ is constructed on a different probability space than the original one. We apply next the Yamada-Watanabe theorem (see, e.g. Theorem 2.1 in [32]) to justify that the existence of the solution on this different probability space together with the pathwise uniqueness of the truncated equation implies the existence of a (unique) solution of the truncated equation on the original space. We have constructed a weakly continuous solution $a^R \in L^2\left( \Omega , L^{\infty }\left( [0,T]; \mathcal {W}^{1,2}(\mathbb {T}^2)^3\right) \right)$. From Lemma 4.1 we can deduce that $\mathbb {E}\left[ \left( \Vert a_t^R\Vert _{1,2}^2 - \Vert a_s^R\Vert _{1,2}^2\right) ^4\right] \le C(t-s)^2$, and therefore by the Kolmogorov-Čentsov criterion, the map $t \rightarrow \Vert a_t^R\Vert _{1,2}^2$ is continuous. Hence $a^R \in L^2\left( \Omega , C\left( 0,T; \mathcal {W}^{1,2}(\mathbb {T}^2)^3\right) \right)$.

The proof of the claim is now complete. $\square$

## Global Solution with Positive Probability

Let $(a, \mathcal {T})$ be a maximal solution of the SRSW system and recall that $\tau ^R = \displaystyle \inf _{t \ge 0}\{\Vert a_t\Vert _{1,2} \ge R\}$. The following technical lemma gives the main estimate for showing the global solution property.

### Lemma 4.1

Let $(a, \mathcal {T})$ be a maximal solution of the SRSW system. Then there exist some positive constants $C_i, i=1,3$, independent of *R* such that

$$\begin{aligned} \Vert a_{t\wedge \tau ^R}\Vert _{1,2}^2 = \Vert a_0\Vert _{1,2}^2 + \displaystyle \int _0^{t\wedge \tau ^R}\tilde{F}(a_s)ds + \displaystyle \sum _{i=1}^{\infty }\displaystyle \int _0^{t\wedge \tau ^R}\tilde{G}_i(a_s)dW_s^i \end{aligned}$$

where $\tilde{F}(a_s)$ and $\tilde{G}_i(a_s)$ are processes such that

$$\begin{aligned} \begin{aligned}&|\tilde{F}(a_s)|^2 \le C_1\Vert a_s\Vert _{1,2}^6 - C_2\Vert a_s\Vert _{1,2}^2 \\&\displaystyle \sum _{i=1}^{\infty }|\tilde{G}_i(a_s)|^2 \le C_2\Vert a_s\Vert _{1,2}^2. \end{aligned} \end{aligned}$$

The proof of this lemma is provided in the Appendix.

### Proposition 4.2

Let $(a, \mathcal {T})$ be a maximal solution of the SRSW system. Then $\tau ^R>0$ $\mathbb {P}$-a.s. for any $R> \Vert a_0\Vert _{1,2}$. In particular $\mathcal {T}>0$ $\mathbb {P}$-a.s.

### Proof

From Lemma 4.1 and the Burkholder-Davis-Gundy inequality we deduce that

$$\begin{aligned} \mathbb {E}\left[ |\Vert a_{t\wedge \tau ^R}\Vert _{1,2}^2 - \Vert a_0\Vert _{1,2}^2|\right] \le tR^6 + \sqrt{t}R^2. \end{aligned}$$

Note that on the set $\{ \tau ^R < t\}$ we have $\Vert a_{t\wedge \tau ^R}\Vert _{1,2} =R$. It follows that

$$\begin{aligned} \begin{aligned} (R^2 - \Vert a_0\Vert _{1,2}^2)\mathbb {P}(\tau ^R< t)&= \mathbb {E}\left[ |\Vert a_{t\wedge \tau ^R}\Vert _{1,2}^2 - \Vert a_0\Vert _{1,2}^2|\mathbb {1}_{\{\tau ^R < t\}}\right] \\&\le \mathbb {E}\left[ |\Vert a_{t\wedge \tau ^R}\Vert _{1,2}^2 - \Vert a_0\Vert _{1,2}^2|\right] \\&\le tR^6+\sqrt{t}R^2. \end{aligned} \end{aligned}$$

Hence

$$\begin{aligned} \mathbb {P}(\tau ^R<t)\le \frac{tR^6+\sqrt{t}R^2}{R^2-\Vert a_0\Vert _{1,2}^2}. \end{aligned}$$

Then

$$\begin{aligned} \displaystyle \lim _{t\rightarrow 0}\mathbb {P}(\tau ^R < t) = 0 \end{aligned}$$

and

$$\begin{aligned} \mathbb {P}(\tau ^R = 0) = \displaystyle \bigcap _{n>0}\mathbb {P}\left( \tau ^R< \frac{1}{n}\right) = \displaystyle \lim _{n\rightarrow \infty }\mathbb {P}\left( \tau ^R < \frac{1}{n}\right) = 0. \end{aligned}$$

Hence $\tau ^R > 0$, $\mathbb {P}$-a.s. and therefore also $\mathcal {T} \ge \tau ^R$ is strictly positive $\mathbb {P}$ almost surely. $\square$

### Proposition 4.3

Let $(a, \mathcal {T})$ be a maximal solution. Then there exists a positive constant *C* such that, if $\Vert a_0\Vert _{1,2} < C$ then $\mathbb {P}(\mathcal {T}=\infty )>0$. In other words, if the initial condition is sufficiently small, then the equation has a global solution.

### Proof

Using the notation in Lemma 4.1, define

$$\begin{aligned} A(a_s)= {\left\{ \begin{array}{ll} \frac{\tilde{F}(a_s)}{\Vert a_s\Vert _{1,2}^2}, &{} \text {if }\ \ a_s \ne 0. \\ 0, &{} \text {if }\ \ a_s=0. \end{array}\right. } \\ B_i(a_s)= {\left\{ \begin{array}{ll} \frac{\tilde{G}_i(a_s)}{\Vert a_s\Vert _{1,2}^2}, &{} \text {if }\ \ a_s \ne 0. \\ 0, &{} \text {if }\ \ a_s=0. \end{array}\right. } \end{aligned}$$

We deduce from Lemma 4.1 that

$$\begin{aligned} \begin{aligned} \Vert a_{t\wedge \tau ^R}\Vert _{1,2}^2&= \Vert a_0\Vert _{1,2}^2 + \displaystyle \int _0^{t\wedge \tau ^R} A(a_s)\Vert a_s\Vert _{1,2}^2 ds + \displaystyle \sum _{i=1}^{\infty }\displaystyle \int _0^{t\wedge \tau ^R} B_i(a_s)\Vert a_s\Vert _{1,2}^2 dW_s^i. \end{aligned} \end{aligned}$$

This implies that

$$\begin{aligned} \begin{aligned} \Vert a_{t\wedge \tau ^R}\Vert _{1,2}^2 = \Vert a_0\Vert _{1,2}^2\exp \left( \displaystyle \int _0^{t\wedge \tau ^R} A(a_s)ds + M_{t\wedge \tau ^R} -\frac{1}{2}[M]_{t\wedge \tau ^R}\right) \end{aligned} \end{aligned}$$

where *M* is the local martingale defined (for $t\ge 0$) as

$$\begin{aligned} M_t = \displaystyle \sum _{i=1}^{\infty }\displaystyle \int _0^tB_i(a_s)dW_s^i \end{aligned}$$

with quadratic variation given by

$$\begin{aligned}{}[M]_t = \displaystyle \sum _{i=1}^{\infty }\displaystyle \int _0^tB_i(a_s)^2ds. \end{aligned}$$

Moreover, since

$$\begin{aligned} \displaystyle \sum _{i=1}^{\infty }|\tilde{G}_i(a_s)|^2 \le C_3\Vert a_s\Vert _{1,2}^2 \end{aligned}$$

we have that

$$\begin{aligned} \displaystyle \sum _{i=1}^{\infty }|B_i(a_s)|^2 \le C_3. \end{aligned}$$

It follows that *M* is a square integrable martingale with quadratic variation $[M]_t \le C_3$. In particular, by Novikov condition, $\exp \left( M_t -\frac{1}{2}[M]_t\right)$ is a martingale and therefore $\mathbb {E}\left[ \exp \left( M_{t\wedge \tau ^R} -\frac{1}{2}[M]_{t\wedge \tau ^R}\right) \right] = 1.$ Next we have from Lemma 6.2 that

$$\begin{aligned} \tilde{F}(a_s) \le c_1 \Vert a_s\Vert _{1,2}^6 - c_2 \Vert a_s\Vert _{1,2}^2 \end{aligned}$$

hence

$$\begin{aligned} A(a_s) \le c_1\Vert a_s\Vert _{1,2}^4 - c_2. \end{aligned}$$

Choose

$$\begin{aligned} \Vert a_0\Vert _{1,2}^2 < \left( \frac{c_2}{c_1}\right) ^{1/4} =: C \end{aligned}$$

and define

$$\begin{aligned} \tau ^C :=\displaystyle \inf _{t} \{ \Vert a_t\Vert _{1,2}^2 \ge C \}. \end{aligned}$$

Then

$$\begin{aligned} \begin{aligned} \mathbb {E}\left[ \Vert a_{t\wedge \tau ^C}\Vert _{1,2}^2\right]&= \Vert a_0\Vert _{1,2}^2\mathbb {E}\left[ \exp \left( \displaystyle \int _0^{t\wedge \tau ^C}A(a_s)ds + M_{t\wedge \tau ^C} - \frac{1}{2}[M]_{t\wedge \tau ^C}\right) \right] \\ {}&< \Vert a_0\Vert _{1,2}^2\mathbb {E}\left[ \exp \left( M_{t\wedge \tau ^C} - \frac{1}{2}[M]_{t\wedge \tau ^C}\right) \right] \\ {}&< C. \end{aligned} \end{aligned}$$

Now

$$\begin{aligned} \begin{aligned} \mathbb {P}\left( \tau ^C = \infty \right)&= \displaystyle \bigcap _N\mathbb {P}(\tau ^C > N) \\ {}&= \displaystyle \lim _{N\rightarrow \infty }\mathbb {P}(\Vert a_{N\wedge \tau ^C} \Vert _{1,2}^2 <C) \\&=\displaystyle \lim _{N\rightarrow \infty }\left( 1-\mathbb {P}\left( \Vert a_{N\wedge \tau ^C} \Vert _{1,2}^2 \ge C\right) \right) \\&\le 1 - \frac{\Vert a_0\Vert _{1,2}^2}{C} \end{aligned} \end{aligned}$$

since we have

$$\begin{aligned} \mathbb {P}(\Vert a_{N\wedge \tau ^C}\Vert _{1,2}^2 \ge C) \le \frac{\mathbb {E} [\Vert a_{N\wedge \tau ^C}\Vert _{1,2}^2]}{C} \le \frac{\Vert a_0\Vert _{1,2}^2}{C} < 1. \end{aligned}$$

It follows that $\mathbb {P}(\tau ^R =\infty ) > 0$ hence the claim. $\square$

## Analytical Properties of the Approximating System

### Relative Compactness

We define the following processes, to shorten the notation:

$$\begin{aligned} \begin{aligned}&X_t^{v^n} := v_0^n + \displaystyle \int _0^t\left( \nu \Delta v_s^n + P_s^{n-1,n}(v_s^n)\right) ds \\ {}&Y_t^{v^n} := \displaystyle \int _0^t \displaystyle \sum _{i=1}^{\infty }[(\mathcal {L}_i + \mathcal {A}_i)v_s^n]dW_s^{i,n}\\ {}&X_t^{h^n} := h_0^n + \displaystyle \int _0^t\left( \eta \Delta h_s^n + Q_s^{n-1,n}(h_s^n)\right) ds \\ {}&Y_t^{h^n} := \displaystyle \int _0^t \displaystyle \sum _{i=1}^{\infty }[\nabla \cdot (\xi _ih_s^n)]dW_s^{i,n}. \end{aligned} \end{aligned}$$

#### Proof of Theorem 3.13

The existence and uniqueness of the solution to the system follows directly from Theorem 6.4. The control (18) holds true from the same theorem and the fact that all coefficients are the same with the exception of the forcing terms, which are bounded uniformly in *n*, as we show below. Let

$$\begin{aligned} F_s^{n-1} = F_s^{n-1,u} + F_s^{n-1,h}:=f_{R}(a_{s}^{n-1}) \left( u_{s}^{n-1} \cdot \nabla v_{s}^{n-1} + \nabla \cdot ( h_{s}^{n-1}u_{s}^{n-1})\right) . \end{aligned}$$

The $L^2$ norm of the first term can be controlled using the truncation and Ladyzhenskaya’s inequality, as follows^4^

$$\begin{aligned} \begin{aligned} \displaystyle \int _0^t\Vert F_s^{n-1,u} \Vert _2^2 ds&=\displaystyle \int _0^t f_{R}(a_{s}^{n-1}) \Vert u_{s}^{n-1} \cdot \nabla v_{s}^{n-1}\Vert _2^2ds \le C\displaystyle \int _0^tf_R(a_s^{n-1})\Vert u_s^{n-1}\Vert _4^2\Vert \nabla v_s^{n-1}\Vert _4^2ds \\&\le CR^3\displaystyle \int _0^t\Vert v_s^{n-1}\Vert _{2,2}ds \le CR^3\sqrt{t}\sqrt{\displaystyle \int _0^t\Vert v_s^{n-1}\Vert _{2,2}^2ds} \\&\le C\sqrt{\tilde{C}}R^3\sqrt{t} \le C_1(R,t). \end{aligned} \end{aligned}$$

Similarly, using Lemma 6.2 from Appendix we have that

$$\begin{aligned} \begin{aligned} \displaystyle \int _0^t\Vert F_s^{n-1,h} \Vert _2^2 ds&= \displaystyle \int _0^tf_R(a_s)\Vert \nabla \cdot (h_s^{n-1}u_s^{n-1}))\Vert _2^2 \le CR^3\displaystyle \int _0^t(\Vert h_s^{n-1}\Vert _{2,2} + \Vert u_s^{n-1}\Vert _{2,2})ds \\&\le 2C\sqrt{\tilde{C}}R^3\sqrt{t} \le C_2(R,T). \end{aligned} \end{aligned}$$

Summing up and using an inductive argument we deduce that there exists a constant *C* which is independent of *n* such that

$$\begin{aligned} \begin{aligned} \mathbb {E}\left[ \Vert a_s^n\Vert _{t,2,2}^2\right]&\le N\left( \Vert a_0\Vert _{1,2}^2 + \mathbb {E}\left[ \displaystyle \int _0^t \Vert F_s^{n-1}\Vert _2^2ds\right] \right) \le N \left( \Vert a_0\Vert _{1,2}^2 + CR^3\sqrt{t}\right) \le C(R,t). \end{aligned} \end{aligned}$$

For an arbitrary $p>2$, we can deduce that there exists a constant $\widetilde{\mathcal {D}}_{p}(T,R)$ such that

$$\begin{aligned} {\mathbb {E}} [\Vert a^n\Vert _{T,2,2}^p ]\le N \left( \Vert a_0\Vert _{1,2}^p + \widetilde{\mathcal {D}}_{p}(T,R) \sqrt{{\mathbb {E}} [ \Vert a^{n-1}\Vert _{T,2,2}^p]} \right) \end{aligned}$$

The result follows with an argument similar to the one used above. For the second part, recall that

$$\begin{aligned} \Vert a_t^n\Vert _{\mathcal {W}^{\beta , p}(0, T ; L^2(\mathbb {T}^2))}^p:=\int _{0}^{T}\left\| a_{t}^n\right\| _{L^2(\mathbb {T}^2)}^{p} d t+\int _{0}^{T} \int _{0}^{T} \frac{\left\| a_{t}^n-a_{s}^n\right\| _{L^2(\mathbb {T}^2)}^{p}}{|t-s|^{1+\beta p}} d t d s. \end{aligned}$$

We show that there exists a constant $C=C\left( T,R\right)$ independent of *n* such that

$$\begin{aligned} E\left[ \left\| a_{t}^{n}-a_{s}^{n}\right\| _{L^2(\mathbb {T}^2)}^{p} \right] \le C |t-s|^{p/2}. \end{aligned}$$

We have

$$\begin{aligned} \begin{aligned} X_t^{v^n}-X_s^{v^n} = \displaystyle \int _s^t P_r^{n-1,n}(v_r^n) dr + \displaystyle \int _s^t\nu \Delta v_r^ndr. \end{aligned} \end{aligned}$$

Then

$$\begin{aligned} \begin{aligned} \mathbb {E}\left[ \Vert X_t^{v^n}-X_s^{v^n}\Vert _2^p \right]&\le \mathbb {E}\left[ \left( \displaystyle \int _s^t \Vert P_r^{n-1,n}(v_r^n)\Vert _2dr\right) ^p + \left( \displaystyle \int _s^t\Vert \Delta v_r^n\Vert _2 dr\right) ^p \right] \\&\le (t-s)^{p}\mathbb {E}\left[ \displaystyle \sup _{r\in [s,t]} \Vert P_r^{n-1,n}(v_r^n)\Vert _2^p \right] \\&\quad + \mathbb {E}\left[ (t-s)^{p/2}\left( \displaystyle \int _0^T\Vert \nu \Delta v_r^n\Vert _2^2dr\right) ^{p/2}\right] \\&\le (t-s)^{p}\mathbb {E}\left[ \displaystyle \sup _{r\in [0,T]} \Vert P_r^{n-1,n}(v_r^n)\Vert _2^p \right] + (t-s)^{p/2}\mathbb {E}\left[ \Vert v_r^n\Vert _{T,2,2}^p\right] \\&\le C(t-s)^{p/2}. \end{aligned} \end{aligned}$$

For the stochastic terms we apply the Burkholder-Davis-Gundy inequality to obtain

$$\begin{aligned} \begin{aligned} \mathbb {E}\left[ \Vert Y_t^{v^n}-Y_s^{v^n}\Vert _2^p \right]&\le \mathbb {E}\left[ \left| \displaystyle \int _s^t \displaystyle \sum _{i=1}^{\infty }\langle v_r^n, (\mathcal {L}_i + \mathcal {A}_i)v_r^n\rangle dW_r^{i,n}\right| ^{p}\right] \\&\le C(p) \mathbb {E}\left[ \displaystyle \int _s^t \displaystyle \sum _{i=1}^{\infty }|\langle v_r^n, (\mathcal {L}_i + \mathcal {A}_i)v_r^n\rangle |^2 dr\right] ^{p/2} \\&\le C(p) \mathbb {E}\left[ \displaystyle \int _s^t\Vert v_r^n\Vert _{2}^2 \displaystyle \sum _{i=1}^{\infty }\Vert (\mathcal {L}_i + \mathcal {A}_i)v_r^n\Vert _{2}^2dr\right] ^{p/2} \\&\le C(p) \mathbb {E}\left[ \displaystyle \int _s^t\Vert v_r^n\Vert _{2}^2 \Vert v_r^n\Vert _{1,2}^2dr\right] ^{p/2} \\&\le C(p)(t-s)^{p/2}\mathbb {E}\left[ \displaystyle \sup _{r\in [s,t]}\Vert v_r^n\Vert _{1,2}^{2p}\right] \\&\le C(p,T)(t-s)^{p/2}. \end{aligned} \end{aligned}$$

With similar arguments

$$\begin{aligned} \begin{aligned} \mathbb {E}\left[ \Vert X_t^{h^n}-X_s^{h^n}\Vert _2^p \right]&\le C(t-s)^{p/2}. \end{aligned} \end{aligned}$$

and

$$\begin{aligned} \begin{aligned} \mathbb {E}\left[ \Vert Y_t^{h^n}-Y_s^{h^n}\Vert _2^p \right]&\le C(t-s)^{p/2}. \end{aligned} \end{aligned}$$

$$\square $$

#### Proposition 5.1

The approximating sequence is relatively compact in the space

$$\begin{aligned} C\left( [0,T], L^2(\mathbb {T}^2)^3\right) . \end{aligned}$$

#### Proof

By a standard Arzela-Ascoli argument (see e.g. [34]), the following compact embedding holds

$$\begin{aligned} L^{\infty }\left( [0,T] , \mathcal {W}^{1,2}(\mathbb {T}^2)^3\right) \cap \mathcal {W}^{\beta ,p}\left( 0,T; L^2(\mathbb {T}^2)^3\right) \hookrightarrow C\left( [0,T], L^2(\mathbb {T}^2) \right) . \end{aligned}$$

This implies that the intersection $B^N:=B^1(0,N)\cap B^2(0,N)$ of any two balls $B^1(0,N) \in L^{\infty }\left( [0,T] , \mathcal {W}^{1,2}(\mathbb {T}^2)^3\right)$ and $B^2(0,N) \in \mathcal {W}^{\beta ,p}\left( 0,T; L^2(\mathbb {T}^2)^3\right)$ is a compact set in the space $C\left( [0,T], L^2(\mathbb {T}^2) \right)$. Observe that

$$\begin{aligned}{} & {} \mathbb {P}\left( a^n \notin B^1(0,N)\right) = \mathbb {P}\left( \displaystyle \sup _{s\in [0,T]}\Vert a_s^n\Vert _{1,2}>N\right) \le \frac{\mathbb {E}\left[ \displaystyle \sup _{s\in [0,T]}\Vert a_s^n\Vert _{1,2}^2 \right] }{N^2} \\{} & {} \quad \mathbb {P}\left( a^n \notin B^2(0,N)\right) = \mathbb {P}\left( \Vert a_s^n\Vert _{\mathcal {W}^{\beta ,p}}>N\right) \le \frac{\mathbb {E}\left[ \Vert a_s^n\Vert _{\mathcal {W}^{\beta ,p}}^p\right] }{N^p}. \end{aligned}$$

Hence

$$\begin{aligned} \begin{aligned} \displaystyle \lim _{N\rightarrow \infty }\displaystyle \sup _n\mathbb {P}\left( a^n \notin B^N\right) \le \displaystyle \lim _{N\rightarrow \infty } \frac{\displaystyle \sup _n\mathbb {E}\left[ \displaystyle \sup _{s\in [0,T]}\Vert a_s^n\Vert _{1,2}^2\right] }{N^2} + \frac{\displaystyle \sup _n\mathbb {E}\left[ \Vert a_s^n\Vert _{\mathcal {W}^{\beta ,p}}^p\right] }{N^p} = 0. \end{aligned} \end{aligned}$$

This justifies the relative compactness of the distribution of $a^n$, that is the tightness of the process $a^n$, provided $\displaystyle \sup _n\mathbb {E}\left[ \displaystyle \sup _{s\in [0,T]}\Vert a_s^n\Vert _{1,2}^2\right] < \infty$ and $\displaystyle \sup _n\mathbb {E}\left[ \Vert a_s^n\Vert _{\mathcal {W}^{\beta ,p}}^p\right] < \infty$. These last two statements are true due to Theorem 3.13 which was proven above. $\square$

## Data Availability

Data sharing not applicable to this article as no datasets were generated or analysed during the current study.

## Appendix

### Lemma 6.1

Let $\left( X^1,\tau ^1\right) , \left( X^2,\tau ^2\right)$ be two local solutions of the SRSW system, and

$$\begin{aligned}{} & {} \bar{X}:=X^1-X^2, \ \ \ \tau ^{1,2} = \tau ^1\wedge \tau ^2, \ \ \ a^i:=\left( X^i, Y^i\right) , \ \ \ \bar{a}:= a^1 - a^2,\\{} & {} \quad Q({\bar{Y}},\bar{X}):=f_R\left( a^1\right) \nabla \cdot \left( Y^1 X^1\right) - f_R\left( a^2\right) \nabla \cdot \left( Y^2X^2\right) \end{aligned}$$

where $\bar{Y}:=Y^1-Y^2$ and *Y* depends linearly on *X*. Then there exists $\zeta >0$ and $C(\zeta ,R)$ such that for $|\alpha |\le k$

$$\begin{aligned} \begin{aligned} |\langle \partial ^{\alpha }\bar{a}, \partial ^{\alpha }Q({\bar{Y}},\bar{X})\rangle |&\le \zeta \Vert \bar{a}\Vert _{k+1,2}^2 + C(\zeta ,R)\Vert Z\Vert \Vert \bar{a}\Vert _{k,2}^2 \end{aligned} \end{aligned}$$

with

$$\begin{aligned} \Vert Z\Vert := C\left( \Vert a^1\Vert _{k,2}^4 + \Vert a^2\Vert _{k,2}^4\right) \end{aligned}$$

### Proof

We use the decomposition

$$\begin{aligned} \begin{aligned} Q(\bar{Y},\bar{X})&= f_R(a^1)\nabla \cdot (Y^1 \bar{X}) + f_R(a^2)\nabla \cdot (\bar{Y} X^2) + |f_R(a^1) - f_R(a^2)| \nabla \cdot (Y^1X^2) \\&:= T_1 + T_2 + T_3. \end{aligned} \end{aligned}$$

$$\square $$

- We have $\nabla \cdot (XY) = X \cdot \nabla Y + Y(\nabla \cdot X) = \mathcal {L}_X Y + \mathcal {D}_X Y$ for any vector *X* and scalar *Y*.
- For $\mathcal {L}_X Y$ use the fact that
  $$\begin{aligned} \begin{aligned} |\langle \partial ^{\alpha }\bar{a}, \partial ^{\alpha }(X \cdot \nabla Y)\rangle |&= |\langle \partial ^{\alpha + 1}\bar{a}, \partial ^{\alpha - 1}(X \cdot \nabla Y)\rangle | \\&\le C \Vert \partial ^{\alpha + 1} \bar{a}\Vert _2\Vert \partial ^{\alpha -1}(X \cdot \nabla Y)\Vert _{2} \\&\le C \Vert \partial ^{\alpha + 1} \bar{a}\Vert _2 \displaystyle \sum _{\beta \le \alpha -1}C\Vert \partial ^{\beta } X\Vert _4\Vert \partial ^{\alpha -\beta } Y\Vert _4 \\&\le C \Vert \partial ^{\alpha + 1} \bar{a}\Vert _2\Vert \partial ^{\beta } X\Vert _2^{1/2}\Vert \partial ^{\beta +1}X\Vert _2^{1/2}\Vert \partial ^{\alpha -\beta } Y\Vert _2^{1/2}\Vert \partial ^{\alpha - \beta +1} Y\Vert _2^{1/2}\\&\le C \Vert \bar{a}\Vert _{k+1,2}\Vert X\Vert _{k-1,2}^{1/2}\Vert X\Vert _{k,2}^{1/2}\Vert Y\Vert _{k,2}^{1/2}\Vert Y\Vert _{k,2}^{1/2} \\&\le C \Vert \bar{a}\Vert _{k+1,2}\Vert X\Vert _{k,2}\Vert Y\Vert _{k,2} \\&\le \frac{\zeta }{2} \Vert \bar{a}\Vert _{k+1,2}^2 + \frac{C_1(\zeta ,R)}{2} \Vert X\Vert _{k,2}^2\Vert Y\Vert _{k,2}^2. \end{aligned} \end{aligned}$$
- Similarly, for $\mathcal {D}_X Y$ use the fact that
  $$\begin{aligned} \begin{aligned} |\langle \partial ^{\alpha }\bar{a}, \partial ^{\alpha }(Y (\nabla \cdot X))\rangle |&= |\langle \partial ^{\alpha + 1} \bar{a}, \partial ^{\alpha - 1}(Y (\nabla \cdot X))\rangle | \\&\le C \Vert \partial ^{\alpha +1} \bar{a}\Vert _2 \displaystyle \sum _{\beta \le \alpha - 1} C \Vert \partial ^{\beta } Y\Vert _4\Vert \partial ^{\alpha - \beta } X\Vert _4 \\&\le \frac{\zeta }{2} \Vert \bar{a}\Vert _{k+1,2}^2 + \frac{C_2(\zeta ,R)}{2} \Vert Y\Vert _{k,2}^2\Vert X\Vert _{k,2}^2. \end{aligned} \end{aligned}$$
- Summing up we have
  $$\begin{aligned} \begin{aligned} |\langle \partial ^{\alpha }\bar{a}, \partial ^{\alpha }(\nabla \cdot (YX)) \rangle | \le \zeta \Vert \bar{a}\Vert _{k+1,2}^2 + C(\zeta ,R)\Vert Y\Vert _{k,2}^2\Vert X\Vert _{k,2}^2. \end{aligned} \end{aligned}$$
- Now apply this for $T_i, i=1,2,3:$
  $$\begin{aligned} \begin{aligned} |\langle \partial ^{\alpha }\bar{a}, \partial ^{\alpha }T_1\rangle |{} & {} \le \frac{\zeta }{3}\Vert \bar{a}\Vert _{k+1,2}^2 + \frac{\tilde{C}(\zeta ,R)}{3}\Vert Y^1\Vert _{k,2}^2\Vert \bar{X}\Vert _{k,2}^2 \\ \end{aligned} \\ \begin{aligned} |\langle \partial ^{\alpha }\bar{a}, \partial ^{\alpha }T_2\rangle |{} & {} \le \frac{\zeta }{3}\Vert \bar{a}\Vert _{k+1,2}^2 + \frac{C(\zeta ,R)}{3}\Vert X^2\Vert _{k,2}^2\Vert \bar{Y}\Vert _{k,2}^2 \end{aligned} \\ \begin{aligned} |\langle \partial ^{\alpha }\bar{a}, \partial ^{\alpha }T_3\rangle |&\le C|f_R(a_1)-f_R(a_2)|\Vert \bar{a}\Vert _{k+1,2}\Vert Y^1\Vert _{k,2}\Vert X^2\Vert _{k,2}\\&\le C\Vert \bar{a}\Vert _{k,2}\Vert \bar{a}\Vert _{k+1,2}\Vert Y^1\Vert _{k,2}\Vert X^2\Vert _{k,2}\\&\le \frac{\zeta }{3}\Vert \bar{a}\Vert _{k+1,2}^2 + \frac{\zeta }{3}\Vert Y^1\Vert _{k,2}^2\Vert X^2\Vert _{k,2}^2\Vert \bar{a}\Vert _{k,2}^2. \end{aligned} \end{aligned}$$

Define

$$\begin{aligned} \begin{aligned} \tilde{Z}&:= C\left( \Vert Y^1\Vert _{k,2}^2 + \Vert X^2\Vert _{k,2}^2 + \Vert Y^1\Vert _{k,2}^2\Vert X^2\Vert _{k,2}^2\right) \le C(\Vert Y^1\Vert _{k,2}^4 + \Vert X^2\Vert _{k,2}^4) \\&\le C(\Vert a^1\Vert _{k,2}^4 + \Vert a^2\Vert _{k,2}^4) \end{aligned} \end{aligned}$$

and

$$\begin{aligned} \Vert Z\Vert :=C(\Vert a^1\Vert _{k,2}^4 + \Vert a^2\Vert _{k,2}^4). \end{aligned}$$

Then

$$\begin{aligned} \begin{aligned} |\langle \partial ^{\alpha }\bar{a}, \partial ^{\alpha }Q({\bar{Y}},\bar{X})\rangle |&\le \zeta \Vert \bar{a}\Vert _{k+1,2}^2 + C(\zeta ,R)\Vert Z\Vert \Vert \bar{a}\Vert _{k,2}^2. \end{aligned} \end{aligned}$$

### Lemma 6.2

The following statements are true: a. There exist some constants $C_1, C_2$ such that for any two vectors *X* and *Y* such that

$Y=\epsilon X + \mathcal {R}$ and for any multi-index $\alpha$ such that $|\alpha | < k$, we have

$$\begin{aligned} |\langle \partial ^{\alpha } Y_t, \partial ^{\alpha }(\mathcal {L}_{X_t}Y_t)\rangle | \le C_1\Vert Y_t\Vert _{k+1,2}^2 + C_2\Vert Y_t\Vert _{k,2}^6. \end{aligned}$$

b. There exist some constants $C_3, C_4, C_5$ such that for any scalar *Y* and vector *X* we have

$$\begin{aligned} |\langle \Delta Y_t, \nabla \cdot (Y_tX_t)\rangle | \le C_3 \Vert \Delta Y_t\Vert _2^2 + C_4\Vert \Delta X_t\Vert _2^{2} + C_5(\Vert Y_t\Vert _{1,2}^6 + \Vert X_t\Vert _{1,2}^6) \end{aligned}$$

### Proof

a. Using Agmon’s, Hölder’s, and Young’s inequalities and the fact that $v = \epsilon u + \mathcal {R}$, we have:

$$\begin{aligned} \begin{aligned} |\langle \partial ^{\alpha } Y_t, \partial ^{\alpha }(\mathcal {L}_{X_t}Y_t)\rangle |&= |\langle \partial ^{\alpha +1} Y_t, \mathcal {L}_{X_t}Y_t\rangle | \le C(\Vert Y_t\Vert _{k+1,2} \Vert X_t\Vert _{\infty }\Vert \partial ^{\alpha } Y_t\Vert _2) \\&\le C(\Vert X_t\Vert _{k+1,2}^{1/2}\Vert X_t\Vert _{k,2}^{1/2}\Vert Y_t\Vert _{k+1,2}\Vert Y_t\Vert _{k,2}) \\&\le C(\Vert Y_t\Vert _{k+1,2}^{1/2}\Vert Y_t\Vert _{k,2}^{1/2}\Vert Y_t\Vert _{k+1,2}\Vert Y_t\Vert _{k,2})\\&\le C(\Vert Y_t\Vert _{k+1,2}^{3/2}\Vert Y_t\Vert _{k,2}^{3/2}) \\&\le C_1\Vert Y_t\Vert _{k+1,2}^2 + C_2\Vert Y_t\Vert _{k,2}^6. \end{aligned} \end{aligned}$$

b. Using Hölder’s, Ladyzhenskaya’s, and Young’s inequalities we have that

$$\begin{aligned} \begin{aligned} |\langle \Delta Y_t, \nabla \cdot (Y_tX_t)\rangle |&= |\langle \Delta Y_t, Y_t\nabla \cdot X_t + X_t\nabla Y_t \rangle | \le \Vert \Delta Y_t\Vert _2\Vert Y_t\nabla X_t\Vert _2 + \Vert \Delta Y_t\Vert _2\Vert X_t \nabla \cdot Y_t\Vert _2 \\&\le C(\Vert \Delta Y_t\Vert _2\Vert Y_t\Vert _4\Vert \nabla X_t\Vert _4 + \Vert \Delta Y_t\Vert _2\Vert X_t\Vert _4 \Vert \nabla Y_t\Vert _4) \\&\le C(\Vert \Delta Y_t\Vert _2\Vert Y_t\Vert _{1,2}\Vert \nabla X_t\Vert _2^{1/2}\Vert \Delta X_t\Vert _2^{1/2} + \Vert \Delta Y_t\Vert _2 \Vert X_t\Vert _{1,2}\Vert \nabla Y_t\Vert _2^{1/2}\Vert \Delta Y_t\Vert _2^{1/2}) \\&\le C(\Vert \Delta Y_t\Vert _2\Vert Y_t\Vert _{1,2}\Vert \nabla X_t\Vert _2^{1/2}\Vert \Delta X_t\Vert _2^{1/2} + \Vert \Delta Y_t\Vert _2^{3/2} \Vert X_t\Vert _{1,2}\Vert Y_t\Vert _{1,2}^{1/2} )\\&\le C( \Vert \Delta Y_t\Vert _{2}^{4/3}\Vert Y_t\Vert _{1,2}^{4/3}\Vert X_t\Vert _{1,2}^{2/3} + \Vert \Delta X_t\Vert _2^2 + \Vert X_t\Vert _{1,2}^6 + \Vert \Delta Y_t\Vert _2^{9/5}\Vert Y_t\Vert _{1,2}^{3/5})\\&\le C(\Vert \Delta Y_t\Vert _2^2 + \Vert Y_t\Vert _{1,2}^4\Vert X_t\Vert _{1,2}^2 + \Vert \Delta X_t\Vert _2^2 + \Vert X_t\Vert _{1,2}^6 + \Vert Y_t\Vert _{1,2}^6 + \Vert \Delta Y_t\Vert _2^2\\&\le C_3 \Vert \Delta Y_t\Vert _2^2 + C_4\Vert \Delta X_t\Vert _2^{2} + C_5(\Vert Y_t\Vert _{1,2}^6 + \Vert X_t\Vert _{1,2}^6). \end{aligned} \end{aligned}$$

$$\square $$

### Proposition 6.3

Assume that $a \in L^2(\Omega , L^2(0,T; \mathcal {W}^{2,2}(\mathbb {T}^2)))$. Then there exists a constant $C=C(R)$ such that

$$\begin{aligned}{} & {} \displaystyle \int _0^t f_R(a_s)^2\Vert \mathcal {L}_{u_s}v_s\Vert _2^2 ds \le C(R) \left[ \displaystyle \int _0^t \Vert v_s\Vert _{2,2}^2 ds+t\right] \\{} & {} \quad \displaystyle \int _0^t f_R(a_s)^2\Vert \nabla \cdot (h_sv_s)\Vert _2^2 ds \le C(R) \left[ \displaystyle \int _0^t \Vert v_s\Vert _{2,2}^2 ds + \displaystyle \int _0^t \Vert h_s\Vert _{2,2}^2 ds + 1\right] . \end{aligned}$$

### Proof

We have

$$\begin{aligned}{} & {} \Vert \mathcal {L}_{u_s}v_s\Vert _2^2 \le \Vert \mathcal {L}_{v_s}v_s\Vert _2^2 + \left( \frac{R}{\epsilon }\right) ^2\Vert \nabla v_s\Vert _2^2 \\{} & {} \quad f_R(a_s)^2\Vert \mathcal {L}_{u_s}v_s\Vert _2^2 \le f_R(a_s)^2\Vert \mathcal {L}_{v_s}v_s\Vert _2^2 + \left( \frac{R}{\epsilon }\right) ^2(R+1)^2 \end{aligned}$$

and

$$\begin{aligned} \begin{aligned} \Vert \mathcal {L}_{v_s}v_s\Vert _2^2&\le \Vert v_s\Vert _4^2\Vert \nabla v_s\Vert _4^2 \le C\Vert v_s\Vert _2\Vert \nabla v_s\Vert _2 \Vert \nabla v_s\Vert _2\Vert \nabla \nabla v_s\Vert _2. \end{aligned} \end{aligned}$$

Hence

$$\begin{aligned} f_R(a_s)^2\Vert \mathcal {L}_{v_s}v_s\Vert _2^2 \le C(R+1)^3\Vert v_s\Vert _{2,2} \end{aligned}$$

and

$$\begin{aligned} \begin{aligned} \displaystyle \int _0^t f_R(a_s)^2\Vert \mathcal {L}_{u_s}v_s\Vert _2^2 ds&\le C(R+1)^3\displaystyle \int _0^t\Vert v_s\Vert _{2,2}ds + \left( \frac{R}{\epsilon }\right) ^2(R+1)^3t \\&\le C(R)\displaystyle \int _0^t(\Vert v_s\Vert _{2,2}^2 + 1)ds. \end{aligned} \end{aligned}$$

Similarly

$$\begin{aligned} \begin{aligned} \Vert \nabla \cdot (h_su_s))\Vert _2^2&\le \Vert \nabla h_s\Vert _4^2\Vert u_s\Vert _4^2 + \Vert h_s\Vert _4^2\Vert \nabla u_s\Vert _4^2 \\&\le C(\Vert \nabla h_s\Vert _2\Vert \nabla \nabla h_s\Vert _2\Vert u_s\Vert _2\Vert \nabla v_s\Vert _2 + \Vert h_s\Vert _2\Vert \nabla h_s\Vert _2\Vert \nabla u_s\Vert _2\Vert \nabla \nabla u_s\Vert _2) \end{aligned} \end{aligned}$$

and

$$\begin{aligned} \begin{aligned} f_R(a_s)\Vert \nabla \cdot (h_su_s))\Vert _2^2 \le C(R+1)^3(\Vert h_s\Vert _{2,2} + \Vert u_s\Vert _{2,2}), \end{aligned} \end{aligned}$$

therefore

$$\begin{aligned} \displaystyle \int _0^t f_R(a_s)^2\Vert \nabla \cdot (h_sv_s)\Vert _2^2 ds \le C(R) \left[ \displaystyle \int _0^t \Vert v_s\Vert _{2,2}^2 ds + \displaystyle \int _0^t \Vert h_s\Vert _{2,2}^2 ds + 1\right] . \end{aligned}$$

$$\square $$

### Proof of Lemma 4.1

One can write

$$\begin{aligned} \begin{aligned}&d\Vert a_{t\wedge \tau ^R}\Vert _{1,2}^2 + 2\gamma \Vert a _{t\wedge \tau ^R}\Vert _{2,2}^2dt \\&= 2\left( \langle \Delta v_{t\wedge \tau ^R}- v_{t\wedge \tau ^R} , Q_{v_{t\wedge \tau ^R}}\rangle + \langle \Delta h_{t\wedge \tau ^R} - h_{t\wedge \tau ^R}, Q_{h_{t\wedge \tau ^R}}\rangle \right) dt\\&\quad + 2 \langle \Delta v_{\wedge \tau ^R}-v_{t\wedge \tau ^R} ,fk\times v_{t\wedge \tau ^R} + g\nabla p_{t\wedge \tau ^R}\rangle dt \\&\quad + \displaystyle \sum _{i=1}^{\infty } \left( \langle (\mathcal {L}_i+\mathcal {A}_i)v_{t\wedge \tau ^R}, (\mathcal {L}_i+\mathcal {A}_i) v_{t\wedge \tau ^R} \rangle _{1,2} + \langle \mathcal {L}_i h_{t\wedge \tau ^R}, \mathcal {L}_i h_{t\wedge \tau ^R}\rangle _{1,2}\right) dt \\&\quad - \displaystyle \sum _{i=1}^{\infty } \left( \langle \Delta v_{t\wedge \tau ^R},(\mathcal {L}_i+\mathcal {A}_i)^2 v_{t\wedge \tau ^R}\rangle + \langle \Delta h_{t\wedge \tau ^R}, \mathcal {L}_i^2 h_{t\wedge \tau ^R}\rangle \right) dt \\&\quad + 2\displaystyle \sum _{i=1}^{\infty }\left( \langle \Delta v_{t\wedge \tau ^R}-v_{t\wedge \tau ^R}, (\mathcal {L}_i+\mathcal {A}_i) v_{t\wedge \tau ^R}\rangle + \langle \Delta h_{t\wedge \tau ^R}-h_{t\wedge \tau ^R}, \mathcal {L}_i h_{t\wedge \tau ^R}\rangle \right) dW_t^i\\ \end{aligned} \end{aligned}$$

where with $Q_v$ and $Q_h$ we denoted, respectively, the corresponding nonlinear parts. Define

$$\begin{aligned} \begin{aligned} \tilde{F}(a_s)&:= 2\left( \langle \Delta v_{s} - v_{s}, Q_{v_{s}}\rangle + \langle \Delta h_{s} - h_{s}, Q_{h_{s}}\rangle + \langle \Delta v_{s} - v_{s} ,fk\times v_{s} + g\nabla p_{s}\rangle \right) \\&\quad + \displaystyle \sum _{i=1}^{\infty } \left( \langle (\mathcal {L}_i+\mathcal {A}_i)v_{s}, (\mathcal {L}_i+\mathcal {A}_i) v_{s} \rangle _{1,2} + \langle \mathcal {L}_i h_{s}, \mathcal {L}_i h_{s}\rangle _{1,2} - \langle \Delta v_{s},(\mathcal {L}_i+\mathcal {A}_i)^2 v_{s}\rangle \right. \\&\quad \left. - \langle \Delta h_{s}, \mathcal {L}_i^2 h_{s}\rangle \right) \\ \tilde{G}_i&:= 2\displaystyle \sum _{i=1}^{\infty }\left( \langle \Delta v_{s}-v_{s}, (\mathcal {L}_i+\mathcal {A}_i) v_{s}\rangle + \langle \Delta h_{s}-h_{s}, \mathcal {L}_i h_{s}\rangle \right) . \end{aligned} \end{aligned}$$

Then by Lemma 6.2 and assumption (6) we have that

25 $$\begin{aligned} \Vert \tilde{F}(a_s)\Vert _{1,2}^2 \le C\Vert a_s\Vert _{1,2}^6 - \zeta \Vert a_s\Vert _{2,2}^2 \le C\Vert a_s\Vert _{1,2}^6 - \zeta \Vert a_s\Vert _{1,2}^2 \end{aligned}$$

since we can choose $\zeta < 2\gamma$ such that (25) holds. Likewise, the control on $\tilde{G}_i$ holds due to assumption (6) and an integration by parts.

The following result is introduced in Theorem 2, pp. 133, in [33], for the *d*-dimensional domain $\mathbb {R}^d$. We rewrite it here for the two-dimensional torus $\mathbb {T}^2$: $\square$

### Theorem 6.4

Suppose that the following conditions hold true:

- $2\sigma ^{ij}(x)\alpha ^i\alpha ^j - \displaystyle \sum _{i=1}^2|\sigma ^{ij}(x)\alpha ^i|^2 \ge b\displaystyle \sum _{i=1}^2|\sigma ^i|^2, \ \ \ \forall \alpha \in \mathbb {T}^2$, where $b>0$ is independent of $t, \omega , x, \alpha$.
- The functions $a^{ij}, b^i, c, \sigma ^{il}, h^l$ with $i,j,l=1,2$ are differentiable in the spatial variable *x* up to order *k*, for all $t, x, \omega$. Moreover, they are uniformly bounded (with respect to all variables) together with their derivatives, by a constant *C*.
- $$u_0 \in L^2(\Omega , \mathcal {W}^{k,2}(\mathbb {T}^2))$$, $$f\in L^2([0,T]\times \Omega ;\mathcal {W}^{k-1}(\mathbb {T}^2))$$, $$g^l \in L^2([0,T]\times \Omega ;\mathcal {W}^{k,2}(\mathbb {T}^2)), l=1,2$$.

Then the generalized solution *u* of the problem

$$\begin{aligned} \begin{aligned}&du(t,x,\omega ) = \left[ (a^{ij}(t,x,\omega )u_i(t,x,\omega ))_j + b^i(t,x,\omega )u_i(t,x,\omega )\right. \\&\quad \left. + c(t,x,\omega )u(t,x,\omega ) + f(t,x,\omega )\right] dt\\&\quad + \left[ \sigma ^{ij}(t,x,\omega )u_i(t,x,\omega ) + h^i(t,x,\omega )u(t,x,\omega ) + g^l(t,x,\omega ) \right] dW_t^l, \ \ \ \\&\quad (t,x,\omega ) \in (0,T]\times \mathbb {T}^2\times \Omega , \end{aligned} \end{aligned}$$

belongs to the class $L^2([0,T],\mathcal {W}^{k+1}(\mathbb {T}^2)) \cap C(0,T; \mathcal {W}^{k,2}(\mathbb {T}^2))$ and there exists $N = N(C,k,T) > 0$ such that

$$\begin{aligned}{} & {} \mathbb {E}\left[ \displaystyle \sup _{t\in [0,T]}\Vert u_t\Vert _{k,2}^2 + \displaystyle \int _0^T \Vert u_t\Vert _{k+1,2}^2dt\right] \\{} & {} \le N\mathbb {E}\left[ \Vert u_0\Vert _{k,2}^2 + \displaystyle \int _0^T\left( \Vert f_t\Vert _{k-1,2}^2 + \displaystyle \sum _{l=1}^2\Vert g_t^l\Vert _{k,2}^2 \right) dt\right] . \end{aligned}$$

## Footnotes

1 We use here the differential notation to match the stochastic version (1).

2 Different levels of viscosity for the different components of *a* can be treated in the same manner.

3 $\mathcal {W} ^{k,2}$ is the standard Sobolev space.

4 Note that *C* can be different at each line.
